# Functional Characterization of Small Alarmone Synthetase and Small Alarmone Hydrolase Proteins from Treponema denticola

**DOI:** 10.1128/spectrum.05100-22

**Published:** 2023-06-08

**Authors:** Miao Wang, Nga-Yeung Tang, Shujie Xie, Rory M. Watt

**Affiliations:** a Faculty of Dentistry, The University of Hong Kong, Pok Fu Lam, Hong Kong SAR, China; b Department of Pathology and Laboratory Medicine, Beaumont Health, Royal Oak, Michigan, USA; c Department of Pathology and Laboratory Medicine, Oakland University William Beaumont School of Medicine, Auburn Hills, Michigan, USA; University of Michigan–Ann Arbor

**Keywords:** oral microbiome, periodontal disease, guanosine tetraphosphate, spirochete, ppApp, enzyme kinetics, nucleotide metabolism

## Abstract

The stringent response enables bacteria to survive nutrient starvation, antibiotic challenge, and other threats to cellular survival. Two alarmone (magic spot) second messengers, guanosine pentaphosphate (pppGpp) and guanosine tetraphosphate (ppGpp), which are synthesized by RelA/SpoT homologue (RSH) proteins, play central roles in the stringent response. The pathogenic oral spirochete bacterium Treponema denticola lacks a long-RSH homologue but encodes putative small alarmone synthetase (Tde-SAS, TDE1711) and small alarmone hydrolase (Tde-SAH, TDE1690) proteins. Here, we characterize the respective *in vitro* and *in vivo* activities of Tde-SAS and Tde-SAH, which respectively belong to the previously uncharacterized RSH families DsRel and ActSpo2. The tetrameric 410-amino acid (aa) Tde-SAS protein preferentially synthesizes ppGpp over pppGpp and a third alarmone, pGpp. Unlike RelQ homologues, alarmones do not allosterically stimulate the synthetic activities of Tde-SAS. The ~180 aa C-terminal tetratricopeptide repeat (TPR) domain of Tde-SAS acts as a brake on the alarmone synthesis activities of the ~220-aa N-terminal catalytic domain. Tde-SAS also synthesizes “alarmone-like” nucleotides such as adenosine tetraphosphate (ppApp), albeit at considerably lower rates. The 210-aa Tde-SAH protein efficiently hydrolyzes all guanosine and adenosine-based alarmones in a Mn(II) ion-dependent manner. Using a growth assays with a Δ*relA*Δ*spoT* strain of Escherichia coli that is deficient in pppGpp/ppGpp synthesis, we demonstrate that Tde-SAS can synthesize alarmones *in vivo* to restore growth in minimal media. Taken together, our results add to our holistic understanding of alarmone metabolism across diverse bacterial species.

**IMPORTANCE** The spirochete bacterium Treponema denticola is a common component of the oral microbiota. However, it may play important pathological roles in multispecies oral infectious diseases such as periodontitis: a severe and destructive form of gum disease, which is a major cause of tooth loss in adults. The operation of the stringent response, a highly conserved survival mechanism, is known to help many bacterial species cause persistent or virulent infections. By characterizing the biochemical functions of the proteins putatively responsible for the stringent response in T. denticola, we may gain molecular insight into how this bacterium can survive within harsh oral environments and promote infection. Our results also expand our general understanding of proteins that synthesize nucleotide-based intracellular signaling molecules in bacteria.

## INTRODUCTION

The “stringent response” is a coordinated set of physiological processes that bacterial cells initiate when they encounter adverse conditions such as nutrient depletion, osmotic shock, or pH extremes. During the stringent response, cellular growth and proliferation are inhibited, and physiological processes that promote cellular survival and persistence are upregulated (reviewed in references 1–8). Two phosphorylated guanine nucleotides, guanosine pentaphosphate (GTP, 3′-diphosphate, pppGpp) and guanosine tetraphosphate (guanosine 3′,5′-bisdiphosphate, ppGpp), are synthesized to millimolar levels during the stringent response (9–13). Colloquially known as “alarmones” or “magic spots,” (p)ppGpp nucleotides function as intracellular second messengers that alter cellular activities at the transcriptional and translational levels, as well as by the direct modulation of enzymatic activities (1–8). More recently, a third alarmone molecule, guanosine 3′-diphosphate, 5-phosphate (pGpp), has also been shown to function as an alarmone in cells (14, 15).

Alarmones are synthesized by RelA/SpoT Homologue (RSH) family proteins (16, 17). Bacteria primarily use multidomain long-RSH (long-Rel) proteins ~700 amino acids (aa) in length to synthesize and/or degrade alarmones. These comprise an N-terminal domain containing SYNTH (synthesis) and HD (His-Asp motif; hydrolysis) subdomains and a C-terminal regulatory domain (16–20). The SYNTH domain catalyzes (p)ppGpp synthesis via the transfer of a diphosphate (PPi, pyrophosphate) group from ATP to the ribose 3′-OH group of GTP/GDP, with the concomitant production of AMP. The HD domain catalyzes (p)ppGpp degradation by hydrolytically removing the 3′-diphosphate group to regenerate GTP/GDP (21–27).

In addition to long-RSH, many bacteria encode monofunctional small alarmone synthetase (SAS) or small alarmone hydrolase (SAH) proteins (16). SASs contain a SYNTH domain, catalyze only alarmone synthesis, and are typically ~200 to 230 aa in length (28–36). SAHs contain an HD domain, are typically 180 to 210 aa in length, and catalyze alarmone hydrolysis (37–39).

The best studied SASs are the respective RelQ and RelP proteins from Staphylococcus aureus, Bacillus subtilis, Streptococcus mutans, and Enterococcus faecalis (RelQ only) (28, 29, 34, 40–42). Other identified SAS lineages include RelV from Vibrio cholerae (30, 43), a dual function RNase HII-(p)ppGpp synthetase from Mycobacterium smegmatis (MS_RHII-RSD) (44, 45), and RelS (Cg2324) from Corynebacterium glutamicum (36). Certain RelQ, RelP, and RelS protein homologues are capable of synthesizing pGpp, albeit to different extents (1, 14, 15, 35, 40, 45, 46).

Recently, certain SAS homologues were shown to synthesize the “alarmone-like” molecules adenosine pentaphosphate (ATP, 3′-diphosphate; pppApp) and adenosine tetraphosphate (adenosine 3′,5′-bisdiphosphate; ppApp). These “toxigenic” SAS (Tox-SAS) proteins and their (p)ppApp products elicited lethal or highly cytotoxic effects within affected cells (1, 31, 47, 48). These Tox-SAS homologues were commonly encoded in toxin-antitoxin (TA) modules alongside a cognate “antitoxin” SAH protein, which had the ability to hydrolyze (p)ppApp to ATP/ADP (31, 38). Certain long-RSH homologues also possess the ability to directly synthesize and/or hydrolyze (p)ppApp (49, 50).

The anaerobic spirochete bacterium Treponema denticola is strongly associated with the development of periodontitis, a chronic infectious-inflammatory disease that affects the gum tissues and underlying tooth-supporting structures (51–55). Periodontitis affects hundreds of millions of people worldwide, and it is the leading cause of tooth loss in adult populations (56). T. denticola is one of ~70 species/species-level phylotypes of Treponema taxa resident in the oral cavity (57, 58).

T. denticola is highly unusual in the bacterial kingdom, as it lacks a long-RSH homologue but encodes a putative SAS homologue (Tde-SAS, TDE1711; 410 aa) and a putative SAH homologue (Tde-SAH, TDE1690; 205 aa) (16, 59). Here, we show that Tde-SAS optimally catalyzes the synthesis of ppGpp over pppGpp and pGpp but also has lower-level ppApp synthesizing activities. We reveal that the presence of the C-terminal TPR motif domain of Tde-SAS represses the synthetic activities of the N-terminal catalytic domain. We further demonstrate that Tde-SAH efficiently hydrolyzes (pp)pGpp and (p)ppApp to the corresponding guanosine- or adenosine-based nucleotides, thus putatively functioning to remove the alarmone (alarmone-like) nucleotides synthesized by Tde-SAS.

## RESULTS

### Domain structure of Tde-SAS.

**FIG 1:**
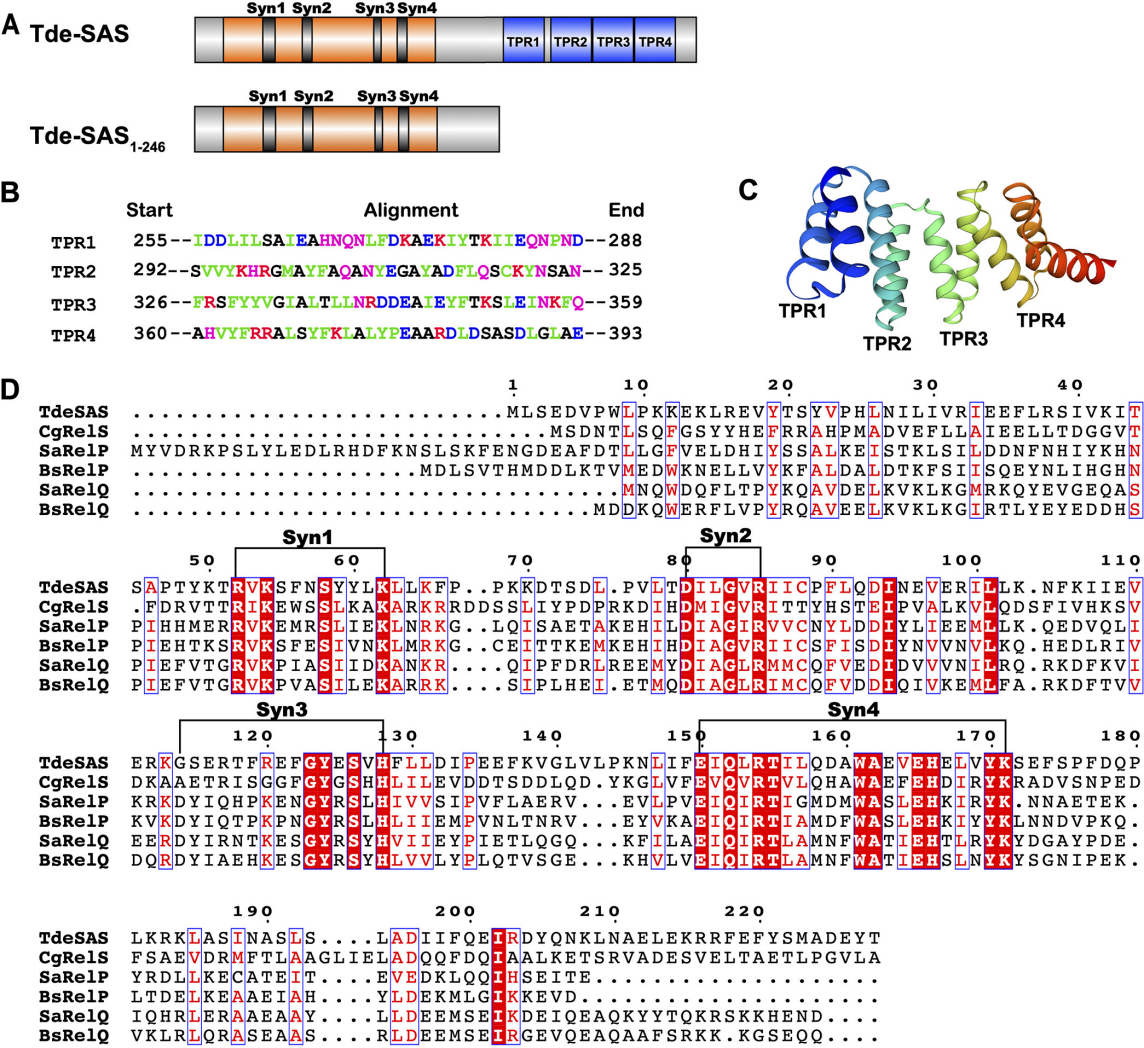
Domain structure and conserved motifs within Treponema denticola encoding putative small alarmone synthetase (Tde-SAS). (A) Schematic diagram showing the domain structure and conserved motifs present within Tde-SAS and Tde-SAS_1–246_ (N-terminal catalytic domain). (B) Sequence alignment of the four tetratricopeptide repeat (TPR) motifs (TPR1 to TPR4) in the C-terminal TPR domain of Tde-SAS, indicating their respective start and end positions, as identified by TPRpred (61). (C) Predicted structural arrangement of the four TPR motifs within the Tde-SAS TPR domain (prepared using SwissModel [90]). The tandem array of four TPR motifs generates a right-handed superhelical structure, with the region immediately C-terminal to TPR4 (colored dark orange/red) forming an extended α-helix. (D) Multiple sequence alignment of the catalytic domain of Tde-SAS (residues 1 to 228 shown) with diverse SAS proteins: C. glutamicum RelS (RelS_Cg_; CgRelS), S. aureus RelP/SAS2 (SaRelP), B. subtilis RelP/Ywac/SAS2 (BsRelP), S. aureus RelQ/SAS1 (SaRelQ), and B. subtilis RelQ/YjbM/SAS1 (BsRelQ). The four respective “synthesis” Syn motifs (Syn1 to Syn4) (27) are indicated with brackets. The figure was prepared using ESPript 3.0 (88).

**FIG 2:**
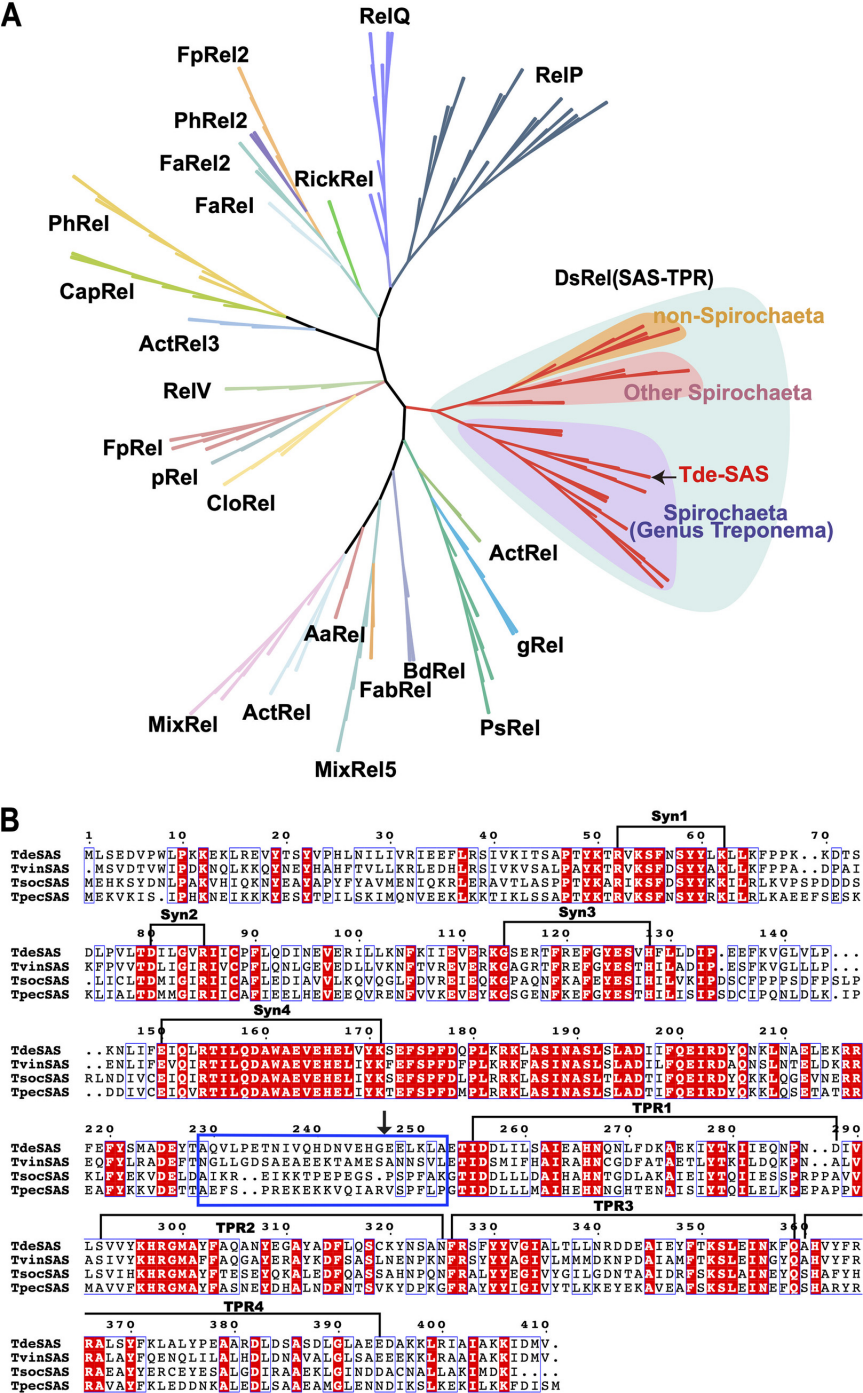
Phylogeny of SAS-TPR (DsRel) homologues and alignment of treponeme SAS-TPR protein sequences. (A) Maximum likelihood (ML) phylogenetic tree of small alarmone synthetase (SAS) homologues, with the major families (phylogenetic clades) indicated. The respective phylogenetic positions of Tde-SAS and other SAS-TPR homologues (DsRel2 family members) from the genus Treponema (shaded violet), from other spirochetes (shaded pink), and from nonspirochete taxa (shaded orange) are indicated. (B) Multiple sequence alignment of Tde-SAS with representative SAS-TPR (DsRel) homologues from Treponema vincentii F0403 (TvinSAS, 409 aa, WP_016518062), Treponema socranskii subsp. *socranskii* ATCC 35536^T^ (TsocSAS, 413 aa, WP_021330562), and Treponema pectinovorum ATCC 700769^T^ (TpecSAS, 415 aa, WP_147612650). The locations of the four synthesis motifs (Syn1 to Syn4) and four TPR motifs (TPR1 to TPR4) are indicated with brackets. The putative linker region is highlighted with a blue box. The C terminus of the Tde-SAS_1–246_ protein is highlighted with an arrow. The SAS/small alarmone hydrolase (SAH) classification system used here was defined as in reference 31. The alignment figure was prepared using ESPript 3.0 (88). The phylograms were prepared using ITOL (94).

The 410-aa Tde-SAS (TDE1711) protein has a distinctive two-domain structure. The N-terminal ~220-aa region shares notable sequence similarity with RelP, RelQ, and RelS (ActRel) homologues and contains all the conserved amino acid residues and structural motifs (Syn1 to Syn4) predicted to be required for alarmone synthesis (Fig. 1A) (27, 41, 42). The C-terminal ~180-aa region contains four tetratricopeptide repeat (TPR) motifs, each of which are predicted to adopt a typical helix-turn-helix conformation (60). Analysis using the TPRpred webserver (61) indicates that these four TPR motifs (TPR1 to TPR4) correspond to residues 255 to 288 (TPR1), 292 to 325 (TPR2), 326 to 359 (TPR3), and 360 to 393 (TPR4), respectively (Fig. 1B and 2). The predicted structural arrangement of the four TPR motifs is shown in Fig. 1C. From here on, we refer to the N-terminal ~220-aa region as the “catalytic domain” and the C-terminal ~180-aa region as the “TPR domain.”

Tde-SAS belongs to the DsRel family of RSH proteins (31), all of which comprise an N-terminal catalytic domain and C-terminal TPR domain. Correspondingly, we refer to these two-domain DsRel-family proteins as “SAS-TPR” homologues (Fig. 2A). SAS-TPR homologues encoded by treponemes (Treponema spp.) share high levels of sequence similarity (Fig. 2B) and form a phylogenetic clade that is distinct from that of SAS-TPR homologues encoded other spirochete taxa or by Deltaproteobacteria (Fig. 2A).

### Biophysical characterization of Tde-SAS.

Recombinant Tde-SAS (molecular weight [MW], 51,318 Da), migrated with an apparent MW of ~220 kDa upon size-exclusion chromatography (SEC) analysis, indicating it formed a stable homotetramer in solution (Fig. S2A). A recombinant protein that comprised only the catalytic domain (Tde-SAS_1–246_; residues 1 to 246; MW, 32,557 Da) migrated with an apparent MW of ~160 kDa upon SEC analysis, suggesting it similarly adopted a homotetrameric arrangement (Fig. S2B). The purified Tde-SAS and Tde-SAS_1–246_ proteins had *A*_260nm_/*A*_280nm_ ratios of 0.71 ± 0.12 and 0.53 ± 0.11, respectively. This indicated that neither protein (obtained by heterologous expression in Escherichia coli) was complexed to RNA, as has previously been observed for long-RSH proteins (62).

The domain structure of Tde-SAS was probed using limited proteolysis with the nonspecific protease subtilisin (18, 63). Two major protein fragments were formed that had apparent MWs of ~26 kDa and ~21 kDa (Fig. S2D). Peptide mass fingerprint (PMF) analysis revealed that these protein fragments respectively corresponded to the catalytic domain and the TPR domain (data not shown). This indicated that subtilisin had primarily cleaved the Tde-SAS protein within the interdomain “linker” region comprising residues ~230 to 250 (Fig. 2B).

### Tde-SAS preferentially synthesizes ppGpp, and its catalytic activities are not notably modulated by alarmones.

**FIG 3:**
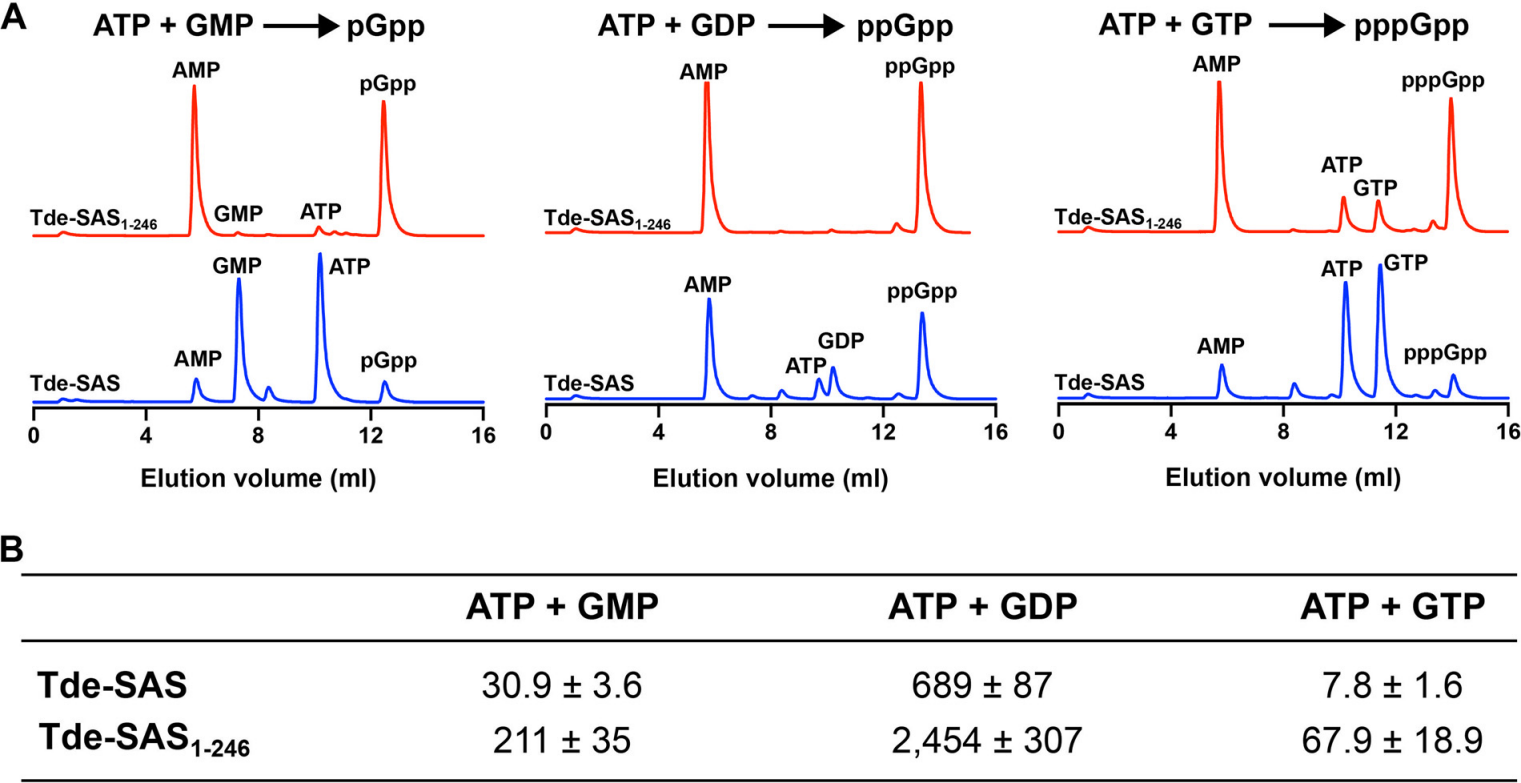
Synthesis of (pp)pGpp by Tde-SAS and Tde-SAS_1–246_. (A) Representative Mono Q anion-exchange chromatograms of enzymatic product mixtures showing the respective (pp)pGpp synthesis activities of Tde-SAS (blue) and Tde-SAS_1–246_ (red) upon incubation with ATP + GMP (left), ATP + GDP (center) or ATP + GTP (right). All sets of assays were performed under analogous conditions. (B) Specific molar activities of the Tde-SAS and Tde-SAS_1–246_ proteins for the synthesis of pGpp (ATP + GMP), ppGpp (ATP + GDP) and pppGpp (ATP + GTP). These rates are reported in the following units: micromoles of (pp)pGpp synthesized per minute per micromole of protein (μmol · min^−1^ · μmol^−1^). All reactions were performed in triplicate, reporting the means ± standard deviation. See Materials and Methods for experimental details.

The results from initial sets of biochemical assays revealed that Tde-SAS catalyzed the synthesis of pppGpp, ppGpp, and pGpp from ATP + GTP, ATP + GDP, and ATP + GMP, respectively, with the concomitant production of AMP. Representative chromatograms of enzymatic product mixtures are shown in Fig. 3A. The optimal pH for ppGpp synthesis activities was approximately 8.8 to 9.2 (Fig. S3A). The specific molar activities of Tde-SAS were determined under standardized conditions to quantify the respective rates of pppGpp, ppGpp, and pGpp synthesis. This was defined in units of micromoles of (pp)pGpp product synthesized per minute per micromole of Tde-SAS protein (μmol · min^−1^ · μmol^−1^) based on monomeric protein concentrations, as previously described (40). The rate of ppGpp synthesis was 689 ± 87 μmol · min^−1^ · μmol^−1^, which was ~22-fold faster than the rate of pGpp synthesis (30.9 ± 3.6 μmol · min^−1^ · μmol^−1^) and ~88-fold faster than the rate of pppGpp synthesis (7.8 ± 1.6 μmol · min^−1^ · μmol^−1^) (Fig. 3B).

Analogous sets of assays were performed for the Tde-SAS_1–246_ protein under identical conditions. Chromatographic analysis of enzymatic product mixtures indicated that Tde-SAS_1–246_ had catalytic activities that were equivalent to those of Tde-SAS but were more potent (Fig. 3A). The respective specific molar rates of pppGpp, ppGpp, and pGpp synthesis by Tde SAS_1–246_ were subsequently determined to be ~3- to 10-fold faster than those of Tde-SAS (Fig. 3B). Both Tde-SAS_1–246_ and Tde-SAS synthesized ppGpp considerably faster than pGpp or pppGpp, suggesting that their substrate utilization patterns were equivalent (i.e., GDP ≫ GMP > GTP).

Micromolar concentrations of Zn^2+^ ions were previously shown to enhance the alarmone-synthesizing activities of Sa-RelP, while Fe^2+^/Fe^3+^ ions played a putative structural role (42). Therefore, we assayed the ppGpp-synthesizing activities of Tde-SAS_1–246_ in the presence of supplementary Fe^3+^ or Zn^2+^ ions (over the concentration range 0 to 32 μM). The results clearly indicated that neither of these metal ions had significant effects on the rate of ppGpp synthesis by Tde-SAS_1–246_ (Fig. S4).

**FIG 4:**
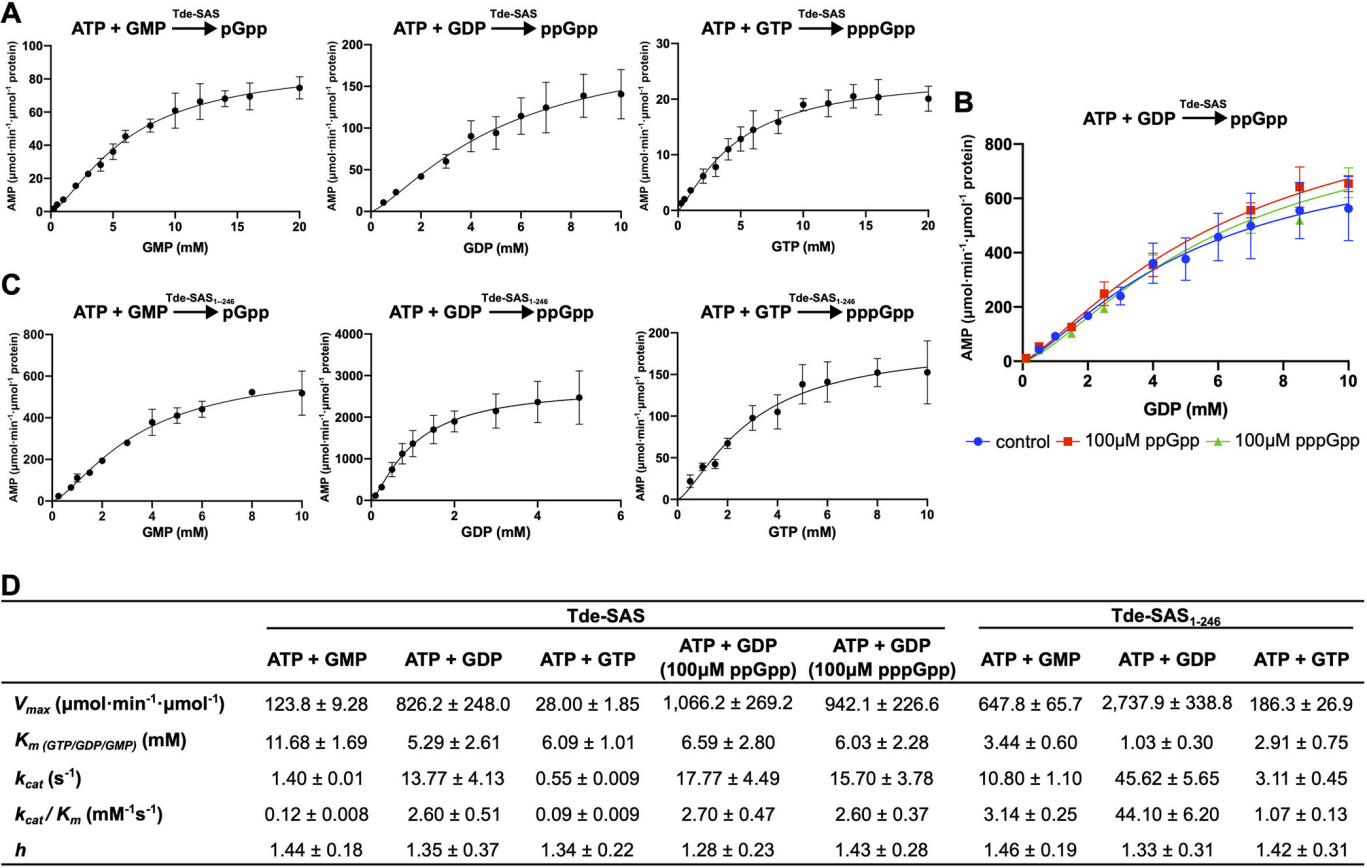
Enzymatic kinetic parameters for (pp)pGpp synthesis by Tde-SAS and Tde-SAS_1–246_ with/without addition of (p)ppGpp. (A) Plots of initial reaction velocity (*V*_0_) versus (GMP, GDP, and GTP) substrate concentration ([S]) used to calculate the kinetic parameters for pGpp (left), ppGpp (center), and pppGpp (right) synthesis by Tde-SAS. The *y* axes show the reaction velocity in units of micromoles of AMP by-product formed per minute per micromole of protein, which is equivalent (equimolar) to the rate of (pp)pGpp synthesis. The *x* axes show substrate concentration (GMP, GDP, or GTP) in millimolar units. (B) Plots of *V*_0_ versus [GDP] used to calculate the kinetic parameters for ppGpp synthesis by Tde-SAS without added ppGpp (control, blue filled circles), in the presence of 100 μM ppGpp (red filled squares), or 100 μM pppGpp (green filled triangles). (C) Plots of V_0_ versus [S] used to calculate the kinetic parameters for pGpp (left), ppGpp (center), and pppGpp (right) synthesis by Tde-SAS_1–246_. (D) Table summarizing the kinetic parameters obtained for (pp)pGpp production by Tde-SAS and Tde-SAS_1–246_ from the respective sets of assays shown in panels A to C: maximum reaction velocity (*V*_max_), Michaelis constants for GTP (*K_m_*_(GTP)_), GDP (*K_m_*_(GDP)_), or GMP (*K_m_*_(GMP)_) in millimolar units; turnover number (*k*_cat_) in units of AMP (= (pp)pGpp) molecules formed per second, enzymatic catalytic efficiency (*k*_cat_/*K_m_*). All reactions were performed in triplicate, reporting the means ± standard deviation. See Materials and Methods for experimental details.

The addition of alarmones have previously been shown to modulate the synthetic activities of RelQ- and RelP-family SAS proteins (14, 40–42, 64). Therefore, the Michaelis-Menten kinetic parameters for ppGpp synthesis were determined for Tde-SAS in the absence of alarmone, as well as in the presence of 100 μM pppGpp or ppGpp (Fig. 4B). In the absence of (p)ppGpp, the *K_m_*_(GDP)_ value was 5.29 ± 2.61 mM, with a corresponding *k*_cat_ value (turnover number) of 13.77 ± 4.13 s^−1^, giving a catalytic efficiency (*k*_cat_/*K_m_*) value of 2.60 ± 0.51 mM^−1^ s^−1^. The Hill coefficient (*h*) was 1.35 ± 0.37, indicating that there may be low levels of positive cooperativity. (Note that *h* > 1 indicates product-mediated stimulation of enzymatic activities, also known as positive cooperativity or feed-forward control.) When 100 μM ppGpp or pppGpp was added, the corresponding *K_m_*_(GDP)_ values increased slightly to 6.59 ± 2.80 and 6.03 ± 2.28 mM, respectively. There was little effect on the corresponding turnover numbers, catalytic efficiencies, or *h* coefficients. These data indicated that added (p)ppGpp had slight stimulatory effects on the catalytic activities of Tde-SAS at a concentration of 100 μM. Additional sets of assays containing 200 μM (allosteric) ppGpp or pppGpp led to only ~10% enhancements in rates of alarmone synthesis (Fig. S5).

As supplementary (p)ppGpp had minor effects on the ppGpp-synthesizing activities of Tde-SAS, the kinetic parameters for pGpp and pppGpp synthesis were determined in the absence of (p)ppGpp (Fig. 4). While the *K_m_* value for GTP (6.09 ± 1.01 mM) was reasonably similar to that for GDP, the *K_m_*_(GMP)_ value was ~2-fold higher (11.68 ± 1.69 mM). The *k*_cat_ values for ppppGpp and pGpp synthesis by Tde-SAS were 25-fold and 10-fold lower than that for ppGpp, respectively. Thus, the catalytic efficiencies for pGpp or pppGpp synthesis by Tde-SAS were ~20- to 30-fold lower than that for ppGpp synthesis. The *h* coefficients for pGpp and pppGpp synthesis were almost identical to that for ppGpp synthesis, indicative of very low-level product-mediated stimulation of enzymatic activities.

### The C-terminal TPR domain inhibits alarmone production.

The respective Michaelis-Menten kinetic parameters for alarmone synthesis by Tde-SAS_1–246_ were determined in the absence of supplementary (p)ppGpp (Fig. 4), as preliminary assays indicated that the addition of 100 μM pppGpp or ppGpp had very minor effects on the rate of ppGpp synthesis (data not shown). The *k*_cat_ value for ppGpp synthesis by Tde-SAS_1–246_ (45.62 ± 5.65 s^−1^) was ~4.5-fold higher than for pGpp synthesis (10.80 ± 1.10 s^−1^) and ~14.5-fold higher than for pppGpp synthesis (3.11 ± 0.45 s^−1^). The *K_m_*_(GTP)_ and *K_m_*_(GMP)_ values were both ~3-fold higher than the *K_m_*_(GDP)_ value. Correspondingly, ppGpp was synthesized with the highest catalytic efficiency (44.10 ± 6.20 mM^−1^ s^−1^), which was ~14-fold higher than that of pppGpp synthesis (3.14 ± 0.25 mM^−1^ s^−1^) and 41-fold higher than that of pGpp synthesis (1.07 ± 0.13 mM^−1^ s^−1^).

The *K_m_*_(GDP)_ value for Tde-SAS_1–246_ (1.03 ± 0.30 mM) was ~5-fold lower than the corresponding value obtained for Tde-SAS (5.29 ± 2.61 mM). The catalytic efficiency for ppGpp synthesis by Tde-SAS_1–246_ was ~18-fold higher than for Tde-SAS (in the absence of added alarmone) (Fig. 4D). Analogously, the *K_m_* values for GMP (3.44 ± 0.6 mM) and GTP (2.91 ± 0.75 mM) for Tde-SAS_1–246_ were, respectively, ~3- and 2-fold lower than the values determined for Tde-SAS. The resultant catalytic efficiencies for pGpp and pppGpp synthesis by Tde-SAS_1–246_ were, respectively, ~26- and 12-fold higher than those of Tde-SAS. Taken together, these results indicated that the presence of the C-terminal TPR domain greatly reduced the overall rates and catalytic efficiencies of (pp)pGpp synthesis but did not greatly affect the substrate preference; GDP was by far the most efficiently utilized substrate.

### Tde-SAS (Tde SAS_1–246_) synthesizes alarmone-like nucleotides (pp)pApp and ppIpp.

**FIG 5:**
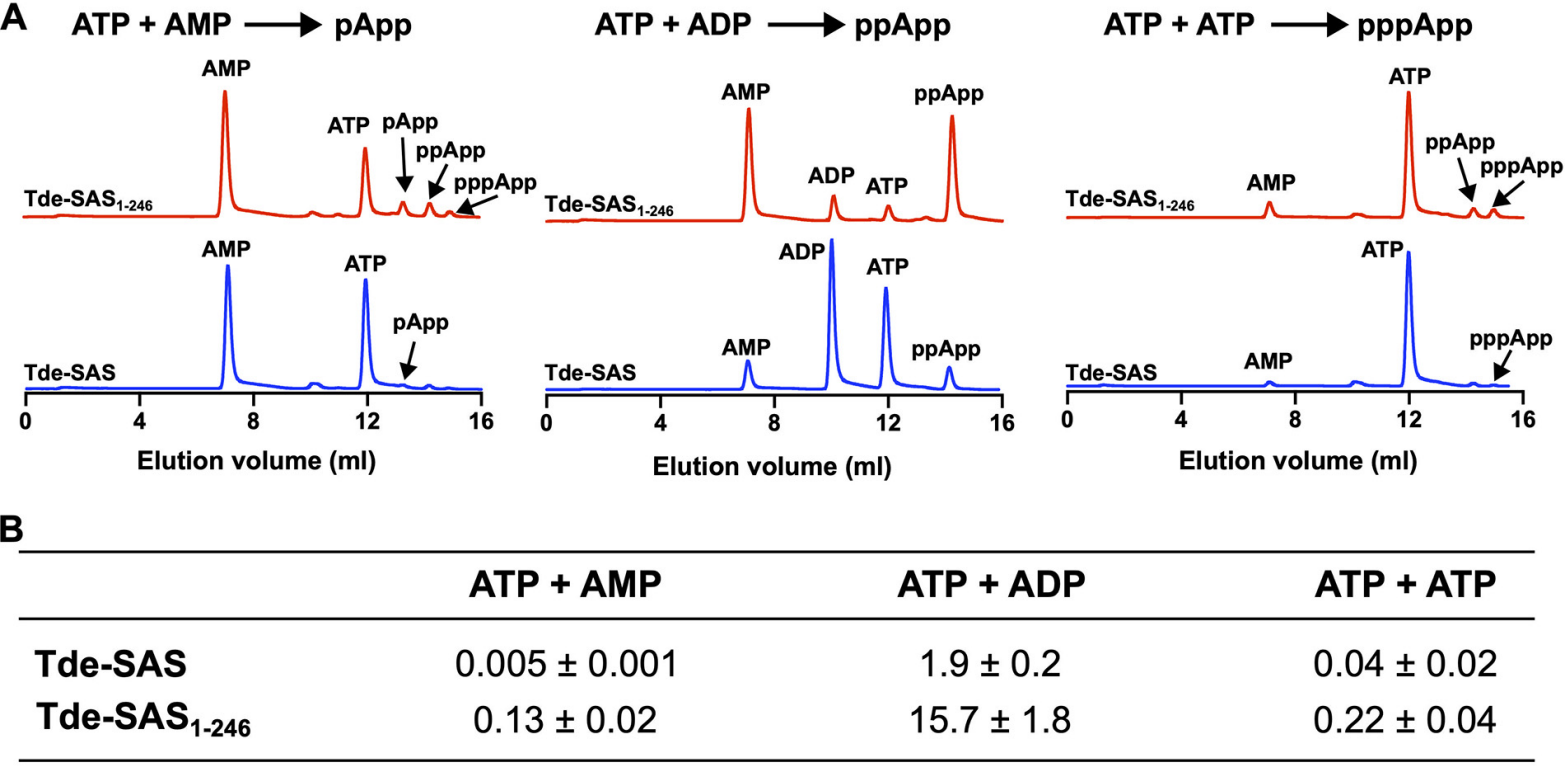
Synthesis of (pp)pApp by Tde-SAS and Tde-SAS_1–246_. (A) Representative Mono-Q anion-exchange chromatograms of enzymatic product mixtures showing the respective (pp)pGpp synthesis activities of Tde-SAS and Tde-SAS_1–246_ upon incubation with ATP + AMP (left), ATP + ADP (center), or ATP (2 equivalents) (right). Blue chromatograms: Tde-SAS, red chromatograms: Tde-SAS_1–246_. All sets of assays were performed under analogous conditions. The corresponding identities of the smaller peaks are indicated with arrows. (B) Specific molar activities of the Tde-SAS and Tde-SAS_1–246_ proteins for the synthesis of pApp (ATP + AMP), ppApp (ATP + ADP), and pppApp (ATP, 2 equivalents). These rates are reported in the following units: micromoles (μmol) of (pp)pGpp or (pp)pApp synthesized per minute per micromole of protein (μmol · min^−1^ · μmol^−1^). All reactions were performed in triplicate, reporting the means ± standard deviation. See Materials and Methods for experimental details.

Tde-SAS and Tde-SAS_1–246_ were incubated with ATP, ATP + ADP, or ATP + AMP, respectively, under standardized conditions to determine their respective abilities to synthesize pppApp, ppApp, and pApp (adenosine 3′-diphosphate, 5′-phosphate). Representative chromatograms of product mixtures are shown in Fig. 5A.

Tde-SAS_1–246_ synthesized ppApp most effectively, with a specific molar activity (15.7 ± 1.8 μmol · min^−1^ · μmol^−1^) that was ~120- and 70-fold higher than that for pppApp and pApp synthesis, respectively (Fig. 5B). Tde-SAS synthesized ppApp ~8-fold slower than Tde-SAS_1–246_, while the rates of pppApp and pApp synthesis were very low under these conditions. The respective rates of ppApp synthesis by Tde-SAS and Tde-SAS_1–246_ were ~160- and 360-fold lower than those of ppGpp synthesis (Fig. 3B). (Note that the additional small (pp)pApp peaks labeled in the chromatograms correspond to the products of competing side reactions, with ppApp primarily formed from the low levels of ADP present in ATP solutions.) Tde-SAS_1–246_ also catalyzed the synthesis of inosine-3′,5′-bis(diphosphate) (inosine tetraphosphate, ppIpp) from IDP and ATP (Fig. S6), as has previously been found for other long-RSH and SAS proteins (18, 40). Tde-SAS exhibited low-level ppIpp synthesis activities under comparable conditions. ITP could not function as a pyrophosphate donor in place of ATP (data not shown).

### Tde-SAH hydrolyzes (pp)pGpp and (p)ppApp in a Mn^2+^ ion-dependent manner.

**FIG 6:**
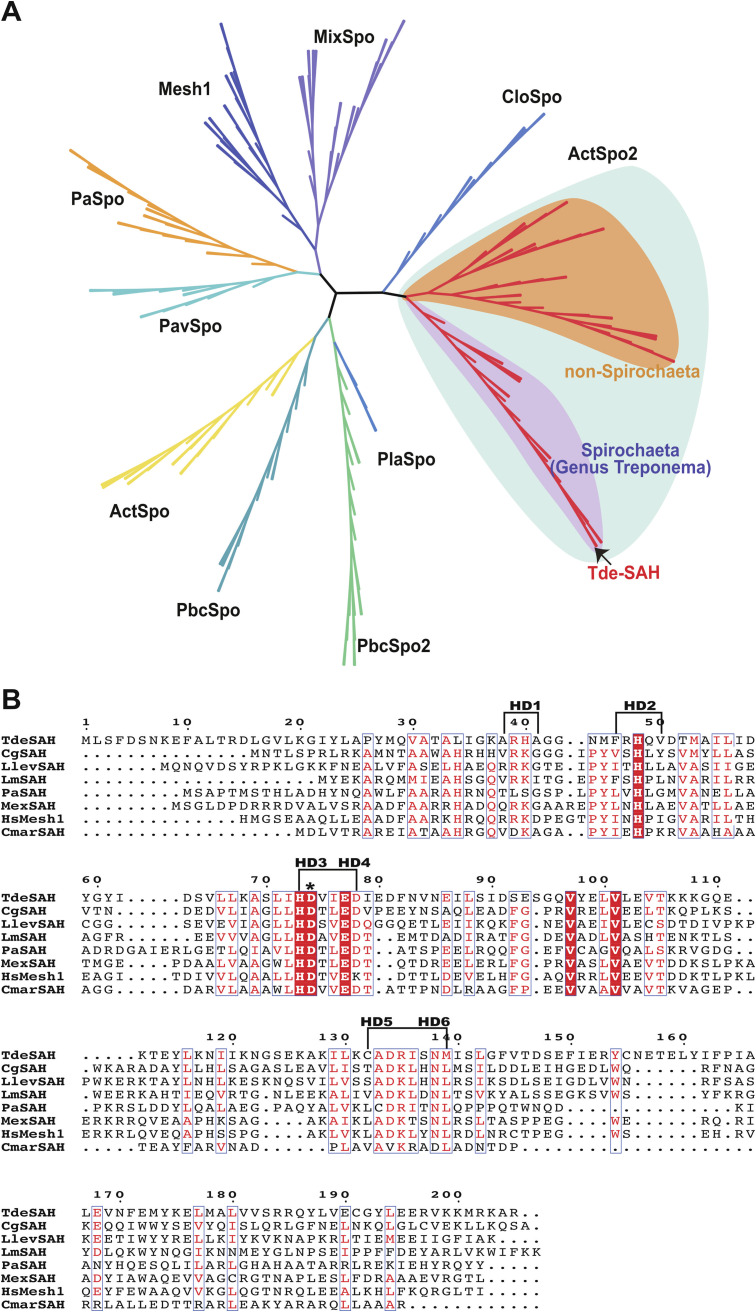
Catalytically important amino acid motifs present within Tde-SAH and representative SAH/Mesh1 homologues. (A) ML phylogenetic tree of SAH homologues, with the major families indicated. The respective phylogenetic positions of Tde-SAH and other SAH homologues belonging to the ActSpo2 family from the genus Treponema (shaded violet) and from nonspirochete taxa (shaded orange) are indicated. (B) Alignment of amino acid sequences of Tde-SAH and representative SAH homologues from diverse families: P. aeruginosa PA0431 (PaSAH), L. levettii RelH (RelH_Ll_; LlevSAH), C. glutamicum RelH (RelH_Cg_; CgSAH), L. monocytogenes Lmo0812 (LmSAH), human MESH1 (HsMESH1) M. extorquens SAH (SAH_Mex_; MexSAH), and C. marina ATFaRel (CmarSAH) proteins. The locations of motifs HD1 to HD6 are indicated with brackets. The location of the Asp74 residue within the eponymous HD domain motif (HD3) is indicated with an asterisk. This residue was mutated to Ala in the Tde-SAH_D74A_ mutant. The figure was prepared using ESPript 3.0 (88).

Tde-SAH (TDE1690) belongs to the ActSpo2 family of RSH proteins (31). Tde-SAH and the other treponeme SAH homologues cluster separately from nontreponeme homologues in the ActSpo2 phylogenetic clade (Fig. 6A), the majority of which belong to taxa from the phylum *Actinobacteria* (31). A multiple sequence alignment of the amino acid sequences of Tde-SAH and seven previously described SAH homologues is shown in Fig. 6B. This includes the C. glutamicum RelH (CgSAH; RelH_Cg_; Protein Data Bank [PDB] entry 7QOD) (36, 39), Listeria monocytogenes Lmo0812 (LmSAH; PDB entry 4YF1), Pseudomonas aeruginosa PA0431 (PaSAH; PDB entry 6YVC) (38), Methylorubrum (Methylobacterium) extorquens SAH_Mex_ (MexSAH) (50), Cellulomonas marina ATFaRel (CmarSAH, WP_090034991) (31), human MESH1 (HsMESH1) (65), and the recently described Leptospira levettii SAH (LlevSAH; RelH_Ll_; PDB entry 7QOE) (39) proteins. The locations of six highly conserved “hydrolase domain motifs” (HD1 to HD6) are indicated (27).

**FIG 7:**
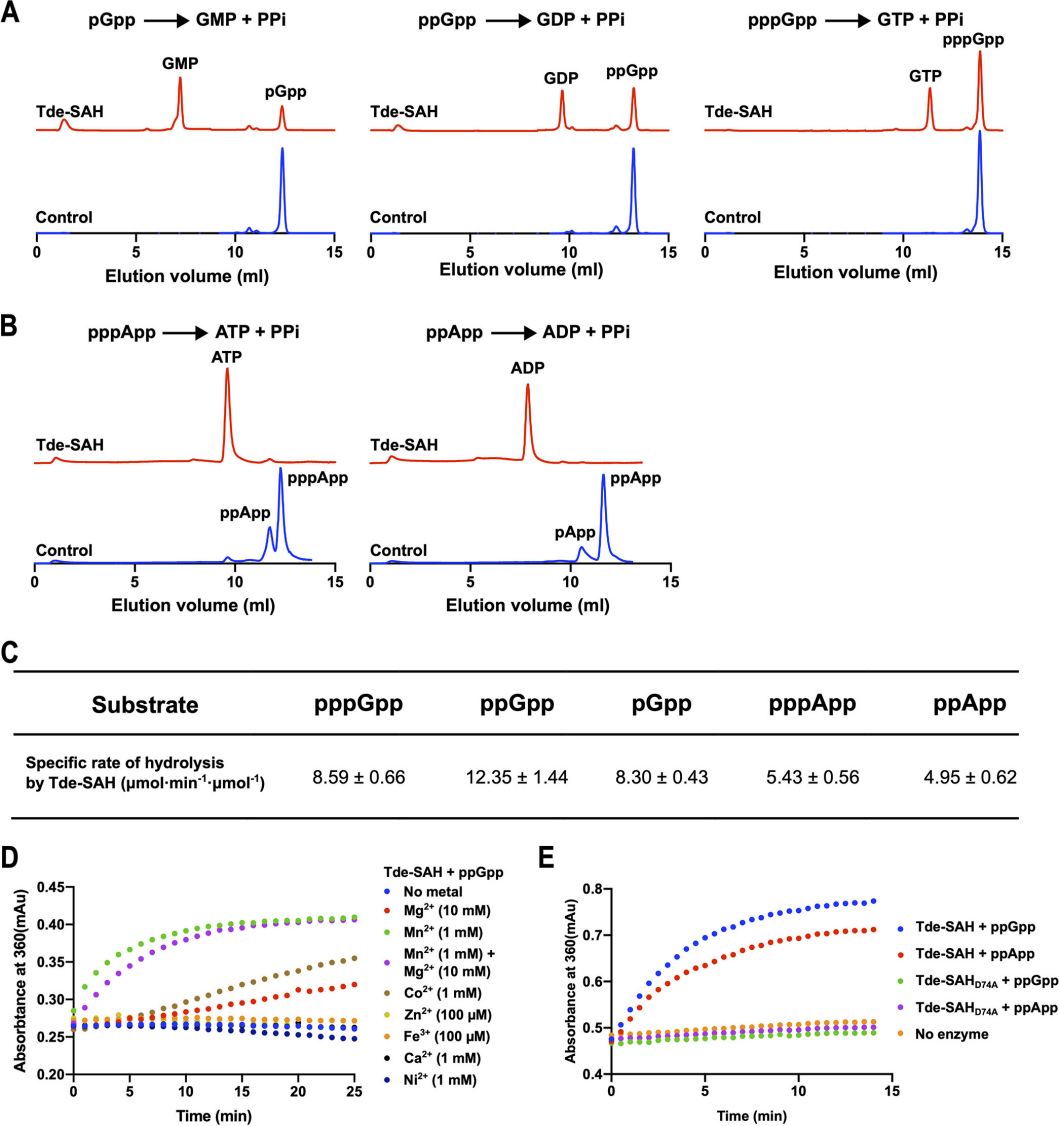
Hydrolysis of (pp)pGpp and (p)ppApp by Tde-SAH. (A) Representative Mono-Q anion-exchange chromatograms of enzymatic product mixtures showing the respective hydrolytic activities of Tde-SAH against pppGpp (left), ppGpp (center), or pGpp (right); 5 min incubation time. (B) Analogous chromatograms showing the respective hydrolytic activities of Tde-SAH against pppApp (left) or ppApp (right); 40 min incubation time. Red chromatograms, Tde-SAH; blue chromatograms, no-added protein (negative) controls. The respective sets of assays shown in panels A and B were performed under analogous conditions. (C) Specific molar activities of Tde-SAH for the hydrolysis of pppGpp, ppGpp, pGpp, pppApp, and ppApp substrates. All reactions were performed in triplicate, reporting the mean, ± standard deviation. (D) Divalent metal ion requirements of Tde-SAH. An overlay is shown of representative plots from continuous spectrophotometric pyrophosphatase-coupled phosphate-release assays quantifying the pyrophosphate released from the hydrolysis of ppGpp by Tde-SAH in buffer containing various metal ions, under standardized conditions. Assays containing 10 mM Mg^2+^ (red), 1 mM Mn^2+^ (green), 10 mM Mg^2+^ and 1 mM Mn^2+^ (magenta), 1 mM Co^2+^ (brown), 100 μM Zn^2+^ (yellow), 100 μM Fe^3+^ (orange), 1 mM Ca^2+^ (black), 1 mM Ni^2+^ (navy blue), and no added metal ions (blue). (E) Alarmone-hydrolyzing activities of Tde-SAH versus Tde-SAH_D74A_ mutant. An overlay is shown of representative plots quantifying the hydrolysis of ppGpp by Tde-SAH (blue), ppApp by Tde-SAH (red), ppGpp by Tde-SAH_D74A_ (green), ppApp by Tde-SAH_D74A_ (magenta), and ppGpp with no enzyme added (orange) in assay mixtures containing 1 mM Mn^2+^ ions under standardized conditions. See Materials and Methods for experimental details.

Recombinant Tde-SAH (MW, 27,091 Da) migrated with an apparent MW of ~44 kDa upon SEC analysis (Fig. S2C), indicating that it was homodimeric, analogous to the RelH_Cg_ and RelH_Ll_ proteins (36, 39). Tde-SAH specifically hydrolyzed pppGpp, ppGpp, and pGpp to form GTP, GDP, and GMP, respectively (Fig. 7A). Tde-SAH hydrolyzed (pp)pGpp in a Mn^2+^ ion-dependent manner. Other (divalent) metal ions were tested as potential cofactors, including Mg^2+^, Co^2+^, Zn^2+^, Fe^3+^, Ca^2+^, and Ni^2+^ (Fig. 7D). Tde-SAH had low-level ppGpp-hydrolyzing activities in the presence of Mg^2+^ ions and slightly higher activities in the presence of Co^2+^ ions but negligible activities when Zn^2+^, Fe^3+^, Ca^2+^, or Ni^2+^ ions were added (Fig. 7D). The hydrolytic activities of Tde-SAH were slightly higher in the presence of 1 mM Mn^2+^ compared to a combination of 1 mM Mn^2+^ and 10 mM Mg^2+^, suggesting that Mn^2+^ and Mg^2+^ ions compete for the same protein-binding sites. The hydrolysis of ppGpp was optimal at pH 8.4 to 8.8 (Fig. S3B). Correspondingly, all subsequent biochemical assays were performed using pH 8.4 buffer containing 1 mM Mn^2+^ ions.

**FIG 8:**
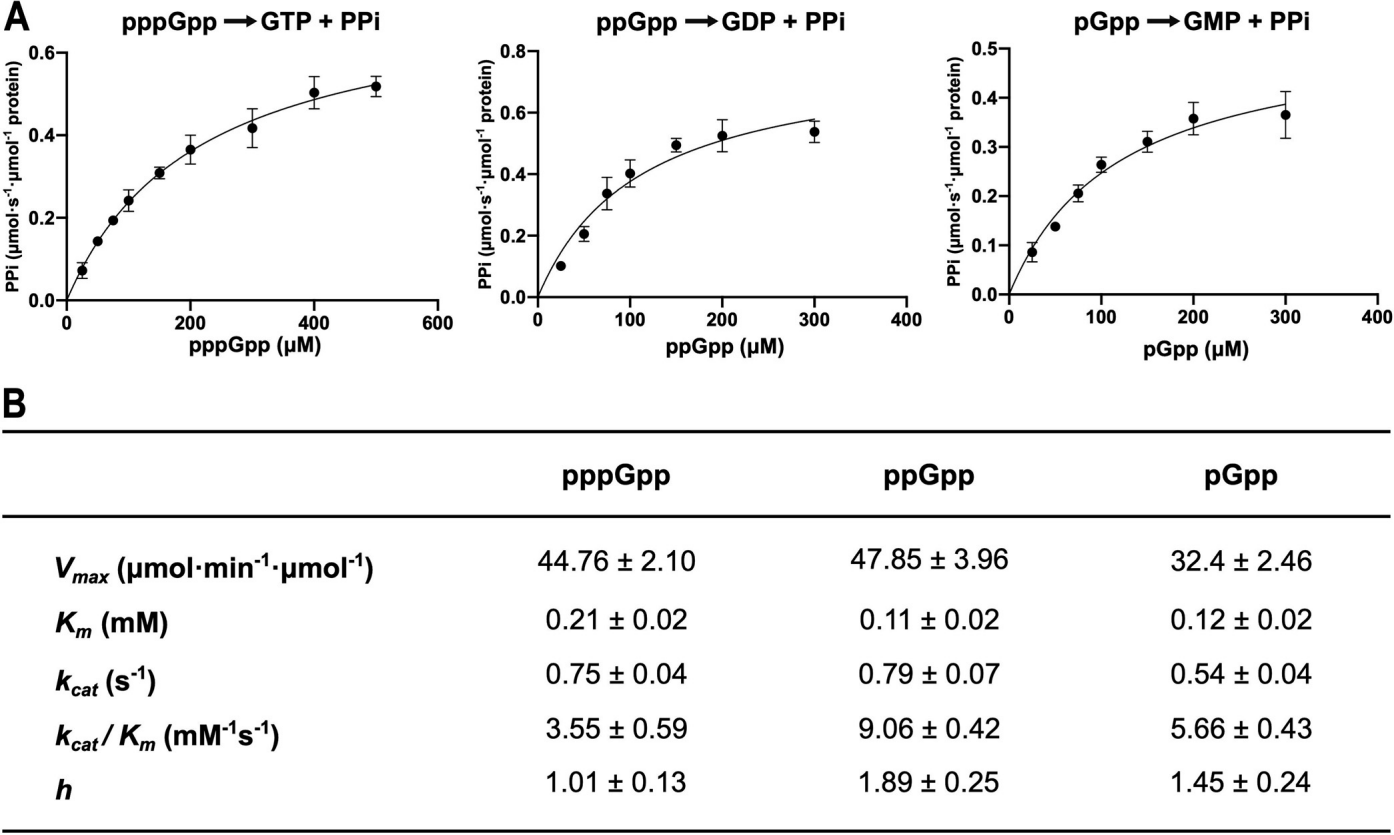
Enzymatic kinetic parameters for (pp)pGpp hydrolysis by Tde-SAH. (A) Plots of initial reaction velocity (*V*_0_) versus substrate concentration ([S]) used to calculate the kinetic parameters for pppGpp (left), ppGpp (center), and pGpp (right) hydrolysis by Tde-SAH. The *y* axes show the reaction velocity in units of micromoles of pyrophosphate (PPi) formed per minute per micromole of protein. The *x* axes show the substrate concentration (pppGpp, ppGpp, pGpp) in micromolar units. (B) Table summarizing the respective kinetic parameters obtained for the hydrolysis of pppGpp, ppGpp, and pGpp by Tde-SAH. Shown are the maximum reaction velocity (*V*_max_) in units of micromoles of pyrophosphate (PPi) formed per minute per micromole of protein; the Michaelis constant (*K_m_*) for the respective pppGpp, ppGpp, or pGpp substrates in millimolar units; and the turnover number (*k*_cat_) in units of pyrophosphate molecules formed per second, enzymatic catalytic efficiency (*k*_cat_/*K_m_*). The data were obtained from sets of continuous spectrophotometric pyrophosphatase-coupled phosphate release assays performed under optimal conditions, in triplicate, reporting the mean values ± standard deviation. See Materials and Methods for experimental details.

Further biochemical analysis using phosphate-release assays with/without the addition of a pyrophosphatase enzyme confirmed that Tde-SAH specifically hydrolyzed the diphosphate (PPi) unit from the 3′-ribose position of (pp)pGpp (data not shown). Consequently, pyrophosphatase enzyme-coupled continuous spectrophotometric assays were performed to determine the Michaelis-Menten kinetic parameters for (pp)pGpp hydrolysis by Tde-SAH, analogous to previously described methods (40). The results indicated that Tde-SAH hydrolyzed pppGpp, ppGpp, and pGpp with similar catalytic efficiencies (Fig. 8). The *k*_cat_ values were in the order ppGpp > pppGpp > pGpp, ranging from 0.79 ± 0.07 to 0.54 ± 0.04 s^−1^. The *K_m_* values were fairly similar for all three alarmones, being lowest for ppGpp and ppGpp at ~100 μM. Correspondingly, Tde-SAH exhibited the highest catalytic efficiency for ppGpp hydrolysis (9.06 ± 0.42 mM^−1^ s^−1^), with pGpp and pppGpp hydrolyzed ~40% and 60% less efficiently, respectively. There was some evidence of positive cooperativity for pGpp and ppGpp hydrolysis, with *h* coefficients of 1.45 ± 0.24 and 1.89 ± 0.25, respectively, but not for pppGpp.

Tde-SAH hydrolyzed pppApp/ppApp, to ATP/ADP + pyrophosphate, respectively, in a Mn^2+^ ion-dependent manner (Fig. 7B). pApp could not be enzymatically synthesized in sufficient quantities and was not tested. It may be noted that under the assay conditions used (pH 8.4, 1 mM Mn^2+^), (p)ppApp molecules are more prone to nonenzymatic (metal ion-facilitated) hydrolysis than (p)ppGpp. Consequently, peaks corresponding to ppApp and pApp are observed in the chromatograms of the control reactions. The specific molar rates of hydrolysis for (pp)pGpp and (p)ppApp were evaluated under identical conditions to quantify the substrate selectivity (Fig. 7C). The results indicated that Tde-SAH hydrolyzed (pp)pGpp ~2-fold faster than pppApp or ppApp under the conditions employed.

The Asp74 residue of Tde-SAH forms part of the HD motif (HD3; Fig. 6B), which has previously been shown to be essential (or critically important) for hydrolytic activity in other RSH homologues (24, 38). The biochemical activities of the Tde-SAH_D74A_ (His-Asp → His-Ala) mutant were determined using assays analogous to those described above. the results revealed that the Tde-SAH_D74A_ protein had undetectable hydrolytic activities against ppGpp or ppApp (Fig. 7E), confirming that Asp74 played an essential role in alarmone hydrolysis.

### Tde-SAS synthesizes alarmones *in vivo*.

The *in vivo* (p)ppGpp synthesis activities of Tde-SAS and Tde-SAS_1–246_ were determined using a well established growth assay in E. coli Δ*relA*Δ*spoT* (CF1693) (23). The CF1693 mutant is deficient in (p)ppGpp synthesis and degradation, exhibits multiple amino acid auxotrophies, and therefore cannot grow in minimal medium (66). Complementation with a plasmid-based RSH protein capable of synthesizing (p)ppGpp to sufficient levels, but not to excessive (i.e., toxic) levels, restores growth in minimal medium (23, 36). The wild-type E. coli MG1655 strain (CF1648) was included as a reference.

**FIG 9:**
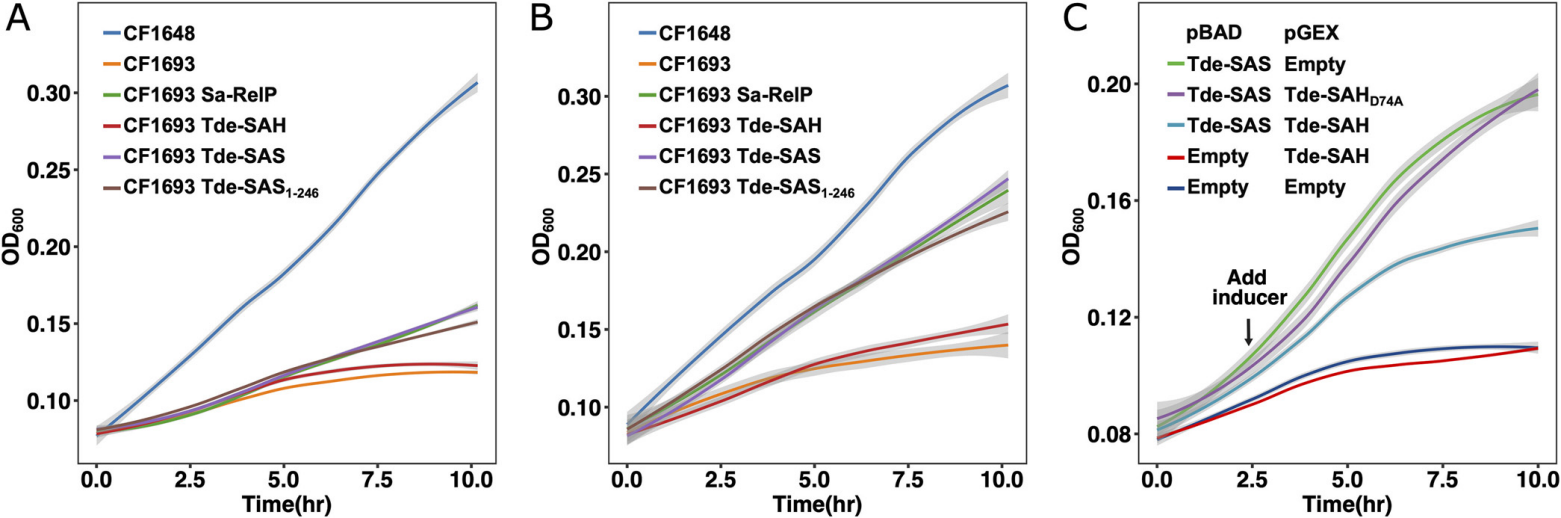
Evaluation of *in vivo* (p)ppGpp synthesis/hydrolysis activities of Tde-SAS, TdeSAS_1–246_, and Tde-SAH. The E. coli Δ*relA*Δ*spoT* (CF1693) strain is deficient in (p)ppGpp synthesis and exhibits negligible growth in minimal medium without complementation with a protein capable of mediating (p)ppGpp synthesis to subtoxic levels. (A, B) Growth curves for CF1693 complemented with (arabinose-inducible) pBAD33-Tde-SAS (purple), pBAD33-Tde-SAS_1–246_ (brown), pBAD33-Tde-SAH (red), and pBAD33-Sa-RelP (green), as well as noncomplemented CF1693 (orange) and wild-type E. coli MG1655 (CF1648, blue). All strains were cultivated in MOPS minimal medium at 37°C. (A) In the absence of arabinose. (B) In the presence of 0.2% arabinose. Growth curves were measured spectrophotometrically (optical density at 600 nm [OD_600_]) over 10 h, plotting the mean value from three biological replicates (shading indicates standard deviation). There were notable enhancements in growth rates for CF1693 strains complemented with pBAD33-Tde-SAS, pBAD33-Tde-SAS_1–246_, and pBAD33-Sa-RelP cultivated in the presence of arabinose, indicative of (p)ppGpp synthesis. (C) Growth curves for CF1693 complemented with pBAD- and pGEX-based plasmids, cultivated in MOPS minimal medium at 37°C. The plasmid combinations tested were pBAD33-Tde-SAS + pGEX-empty (green), pBAD33-Tde-SAS + pGEX-Tde-SAH_D74A_ (purple), pBAD33-Tde-SAS + pGEX-Tde-SAH (light-blue), pBAD33-empty + pGEX-Tde-SAH (red), and pBAD33-empty + pGEX-empty (dark-blue). Expression was induced by the addition of isopropyl β-d-1-thiogalactopyranoside (IPTG; 0.2 mM) and arabinose (0.2%) after 2.5 h (arrow). The growth rates were highest for the pBAD33-Tde-SAS + pGEX-empty and pBAD33-Tde-SAS + pGEX-Tde-SAH_D74A_ strains. The growth rates of the strains complemented with pBAD33-empty + pGEX-Tde-SAH and pBAD33-empty + pGEX-empty were very low. The growth rate of the pBAD33-Tde-SAS + pGEX-Tde-SAH strain was intermediate. See Materials and Methods for detailed experimental protocols.

Genes encoding the Tde-SAS, Tde-SAS_1–246_, Tde-SAH, and S. aureus RelP (Sa-RelP) proteins were respectively cloned into the medium-copy number and arabinose-inducible pBAD33 plasmid (67) and established in the E. coli Δ*relA*Δ*spoT* strain. The pBAD33-Sa-RelP and empty pBAD33 plasmids were included as positive and negative controls, respectively. Cultivation in MOPS minimal medium lacking arabinose led to modest increases in growth rates for the pBAD33-Tde-SAS, pBAD33-Tde-SAS_1–246_, and pBAD33-Sa-RelP complemented strains, compared to the pBAD33-Tde-SAH and pBAD33 strains (Fig. 9A). Analogous experiments performed in MOPS minimal medium containing arabinose (0.2%) led to notable increases in growth rates for the strains complemented with the pBAD33-Tde-SAS, pBAD33-Tde-SAS_1–246_, and pBAD33-Sa-RelP plasmids, indicative of (p)ppGpp production to subtoxic levels (Fig. 9B). In contrast, there were negligible changes in the growth rates of the strains containing the empty pBAD33 and pBAD33-Tde-SAH plasmids. The growth rate of the wild-type CF1648 (MG1655) strain complemented with an empty pBAD33 plasmid remained essentially unchanged in absence and presence of inducer, which were considerably higher than those of all the complemented Δ*relA*Δ*spoT* strains under all conditions employed. Taken together, these results strongly support the premise that both the Tde-SAS and Tde-SAS_1–246_ proteins can synthesize (p)ppGpp in the E. coli Δ*relA*Δ*spoT* strain to levels that can maintain its effective growth in minimal medium.

### Tde-SAH can hydrolyze (p)ppGpp produced by Tde-SAS *in vivo*.

Further sets of growth complementation experiments were performed to investigate the *in vivo* alarmone-hydrolyzing activities of Tde-SAH, as well as the Tde-SAH_D74A_ mutant. In these experiments, two plasmids were stably coestablished in E. coli Δ*relA*Δ*spoT*: (i) a high-copy number, isopropyl β-d-1-thiogalactopyranoside (IPTG)-inducible pGEX plasmid (Amp^R^) containing the Tde-SAH wild-type or Tde-SAH_D74A_ mutant genes or an empty pGEX plasmid and (ii) a pBAD33 (Cm^R^) plasmid containing the Tde-SAS gene or an empty pBAD33 plasmid. Growth in MOPS minimal medium was measured over 10 h at 37°C, adding both the IPTG (0.2 mM) and arabinose (0.2%) inducers at the 2.5-h point. Growth curves are shown in Fig. 9C.

The E. coli Δ*relA*Δ*spoT* strains complemented with the pBAD33-Tde-SAS + pGEX-empty and the pBAD33-Tde-SAS + pGEX-Tde-SAH_D74A_ pairs of plasmids grew most effectively. This supported the premise that the Tde-SAH_D74A_ mutant could not hydrolyze the (p)ppGpp synthesized by Tde-SAS. Complementation with the pBAD33-empty + pGEX-empty or the pBAD33-empty + pGEX-Tde-SAH plasmids resulted in similarly low growth rates. However, complementation with pBAD33-Tde-SAS + pGEX-Tde-SAH led to growth rates intermediate between these two levels. Taken together, these results suggest that Tde-SAS synthesized sufficient quantities of (p)ppGpp, which survived long enough within the cell to mediate various growth-promoting stringent response effects before being degraded by Tde-SAH (Fig. 9C).

## DISCUSSION

To the best of our knowledge, there have been no investigations into the operation of the stringent response in T. denticola or any other Treponema species, and its biological significance remains unexplored. T. denticola is remarkable by encoding one SAS and one SAH protein but lacking a bifunctional long-RSH (long-Rel) homologue (16, 31, 59). Long-RSH proteins appear to be present in all other Treponema species, with the notable exceptions of Treponema pallidum, Treponema putidum, and Treponema pedis (Table S3) (16, 31, 68–70). Our results here indicate that Tde-SAS (TDE1711) is putatively the source of (pp)pGpp and (p)ppApp in T. denticola, and Tde-SAH (TDE1690) is the protein responsible for (pp)pGpp and (p)ppApp degradation.

More than 20 different families (phylogenetic lineages) of SAS proteins have been identified (31), but only a small number have yet been studied. Tde-SAS represents the first protein in the DsRel (SAS-TPR) family to be functionally characterized. Below, we compare and contrast the characteristics and biochemical activities of Tde-SAS with homologues from other SAS families.

Tde-SAS (and Tde-SAS_1–246_) adopts a homotetrameric arrangement analogous to RelP and RelQ homologues from *Firmicutes* (40–42, 64, 71). Similar to RelP and C. glutamicum RelS (ActRel_Cg_), Tde-SAS lacks the consensus **K**XKX**K**XX**R** (R1), **EF**V**T** (R2), and LAM**N**FWAT (R3) motifs present in RelQ homologues, which are responsible for the allosteric binding of (p)ppGpp molecules that stimulate (p)ppGpp synthesis activities (14, 40–42, 64). This is consistent with the synthetic activities of Tde-SAS not being notably stimulated by the addition of (p)ppGpp (Fig. 5). Furthermore, unlike S. aureus RelP, added (p)ppGpp did not inhibit Tde-SAS activities, and micromolar concentrations of Zn^2+^ ions do not stimulate its activities (41, 42). Tde-SAS lacks potential metal-chelating residues equivalent to the adjacent histidine residues (His73 and His74) of S. aureus RelP that bind Zn^2+^ ions at the dimer interface, which putatively modulate its catalytic activities in response to changes in oxidative stress (42). This highlights the subtle differences in modulatory mechanisms operating within different SAS protein homologues.

Tde-SAS preferentially utilizes GDP for the synthesis of ppGpp. Broadly speaking, this is similar to the majority of RelP and RelQ homologues studied to date, under the majority of experimental conditions tested (14, 29, 32, 40–42, 64). C. glutamicum RelS synthesizes pppGpp most efficiently but exhibits complex kinetic behaviors (36). The bifunctional RNase-SAS RelZ protein from M. smegmatis has been reported to synthesize pGpp most efficiently (45). The recently described RelQ homologue from Clostridiodes (Clostridium) difficille (RelQ_Cd_) is a notable outlier, having the ability to utilize GDP and GTP for the production of pGpp, putatively via an intermediate phosphohydrolase step (35). We found no evidence that Tde-SAS catalyzes any competing phosphotransferase or phosphohydrolase processes throughout our experimental work.

The *K_m_*_(GDP)_ value for Tde-SAS (5.29 ± 2.61 mM) is similar to that of C. glutamicum RelS (6.1 mM) (36) but is considerably higher than the values previously reported for M. smegmatis RelZ and *Firmicutes* RelP and RelQ homologues, which are in the range 0.1 to 1.7 mM (14, 40, 41, 45, 64). However, removal of the TPR domain (as in the Tde-SAS_1–246_ protein) dramatically increased (pp)pGpp production levels. It increased the rate of (pp)pGpp synthesis ~3-fold and reduced *K_m_*_(GDP)_ more than 6-fold (to ~1 mM), making it comparable to the above-mentioned SAS proteins. Putatively, this would greatly increase ppGpp production at GDP concentrations typically found within bacterial cells (13, 46, 72–74). However, removing the TPR domain did not alter substrate selectivity, with GDP strongly preferred over GTP or GMP. Thus, the TPR domain appears to act like a brake, repressing the overall alarmone-synthesizing activities of the Tde-SAS catalytic domain by reducing the effective substrate binding affinity (increasing *K_m_*) and reducing the overall reaction rate (decreasing *k*_cat_).

Both Tde-SAS and Tde-SAS_1–246_ were capable of synthesizing sufficient quantities of (p)ppGpp to restore the growth of the E. coli Δ*relA*Δ*spoT* (CF1693) strain in minimal medium, without inducing toxic effects (due to the overproduction of (p)ppGpp) (Fig. 9). While these sets of experiments demonstrated *in vivo* alarmone synthesis activities in a model bacterial system, they do not directly show that Tde-SAS (or Tde-SAS_1–246_) synthesizes alarmones within the native T. denticola host. It should also be noted that this E. coli reporter system is semiquantitative in nature and cannot be used to accurately evaluate levels of alarmone production or hydrolysis. These are notable limitations of our study.

Proteins containing TPR motif domains are widely distributed throughout prokaryotes, in which they typically function as protein “interaction modules” that promote the formation of multiprotein complexes, function as chaperones, or regulate the extracellular export of proteins or exopolysaccharides (60, 75–79). While TPR motif domains comprise multiple (typically 3 to 16) tandem repeats of a 34-aa structural motif that adopts a distinctive helix-turn-helix conformation, there is considerable sequence heterogeneity in their sequence composition (60, 75). There are high levels of sequence conservation within the four respective TPR motifs in SAS-TPR homologues encoded by diverse Treponema species, indicative of conservation of structure and/or function (Fig. 2). We speculate that the TPR domain of Tde-SAS and other treponeme SAS-TPR (DsRel) homologues play analogous regulatory roles in the stringent response (see below).

Tde-SAS synthesized three different adenine nucleotide-based alarmone products: pppApp, ppApp, and pApp, albeit at greatly differing rates (Fig. 5). The pattern for AMP/ADP/ATP utilization (as diphosphate acceptor) was equivalent to that of GMP/GDP/GTP, i.e., ADP was strongly preferred for ppApp synthesis, just as GDP was preferred for ppGpp production. Notably, the rates of (pp)pApp production were tens to hundreds of times lower than the corresponding rates of (pp)pGpp production. Tde-SAS_1–246_ synthesized ppApp ~8-fold faster than Tde-SAS, indicating that the TPR domain of Tde-SAS repressed (pp)pGpp and (pp)pApp production by the catalytic domain via the same mechanism.

The physiological functions and molecular sources of (p)ppApp within bacterial cells remains poorly understood (1, 31, 38, 46, 47, 49, 80). Several phylogenetic lineages of SAS proteins (PhRel, CapRel, PhRel2, and FaRel) are prolific (p)ppApp synthesizers, leading to potent cytotoxic effects (31, 47). In addition to functioning as toxins, (p)ppApp directly modulates enzymatic activities (e.g., PurF) and alters transcriptional activities in a manner notably different from that of (p)ppGpp (47, 81–83). Thus far, Rel_Mex_ is the only long-RSH protein shown to possess notable (p)ppApp synthesis activities (49, 50). The majority of SAS and Rel (long-RSH) proteins characterized to date do not appear to function as sources of (p)ppApp (1, 5, 38, 46, 49, 50). The putative *in vivo* ppApp-synthesizing abilities of Tde-SAS remain to be verified. However, Tde-SAS synthesizes ppApp rather slowly *in vitro*, and results from our growth rate assays indicate that Tde-SAS does not function as a “Tox-SAS” in E. coli. Furthermore, the presence of (pp)pApp in T. denticola cells remains unknown. It is conceivable that Tde-SAS synthesizes low levels of (p)ppApp as an alarmone-like transcriptional modulator or for other regulatory purposes in T. denticola cells (3, 81–83). In these regards, our study has notable limitations, and these important issues require future investigation.

There are many distinct phylogenetic lineages (families) of SAH proteins (31) (Fig. 6A), several of which have been functionally and/or structurally characterized. Tde-SAH represents the first homologue from the ActSpo2 family to be functionally characterized. The Metazoan SpoT homologue 1 (Mesh1) family, which is distributed across bacteria and metazoans, includes human MESH1 (PDB entry 3NR1; HsMesh1), Drosophila melanogaster Mesh1 (PDB entry 3NQW) (65, 84) and M. extorquens SAH (SAH_Mex_; MexSAH) (50). The MixSpo (RelH) family includes C. glutamicum RelH_Cg_ (PDB entry 7QOD; CgSAH) (37, 39), L. levettii RelH_Ll_ (PDB entry 7QOE; LlevSAS) (39), and L. monocytogenes Lmo0812 (PDB entry 4YF1; LmSAH). The PbcSpo2 family includes P. aeruginosa SAH (PA0431; PDB entry 6YVC; PaSAH) (38), and the PbcSpo family includes C. marina ATFaRel (CmarSAH) (31).

Tde-SAH hydrolyzed pppGpp/ppGpp/pGpp to GTP, GDP, or GMP + pyrophosphate, respectively, with comparable catalytic efficiencies, dependent on Mn^2+^ ions (Fig. 7). Low-level hydrolytic activities were supported by Mg^2+^ ions or Co^2+^ ions. Tde-SAH hydrolyzed (p)ppApp to ATP/ADP + PPi in an analogous manner, at roughly equivalent rates. Thus, the hydrolytic activities of Tde-SAH functionally complement the synthetic activities of Tde-SAS. The respective catalytic efficiencies and *K_m_* values for alarmone hydrolysis by Tde-SAH [*K_m_*_(pppGpp)_, 210 μM; *K_m_*_(ppGpp)_, 110 μM; *K_m_*_(pGpp)_, 120 μM] are very similar to those of the RelH_Cg_ SAH protein [*K_m_*_(pppGpp)_, 90 μM; *K_m_*_(ppGpp)_, 101 μM; *K_m_*_(pGpp)_, ~140 μM] (37). The (p)ppApp hydrolysis activities of RelH_Cg_ and RelH_Ll_ remain unexplored (39). SAH_Mex_ had negligible hydrolytic activities toward (p)ppGpp substrates but efficiently hydrolyzed pppApp and ppApp substrates (50). Drosophila Mesh1 hydrolyzed (p)ppApp and (p)ppGpp substrates with similar catalytic efficiencies, with *K_m_* values ranging from 60 to 120 μM. While Mesh1 homologues probably function as (p)ppApp and/or (p)ppGpp hydrolases in bacteria, they are proposed to function primarily as NADPH phosphatases in metazoans (50, 84, 85). The PaSAH protein showed a notable preference for adenine-based alarmones, hydrolyzing (p)ppApp ~5- to 10-fold more rapidly and more efficiently than (p)ppGpp (38, 50). Taken together, SAH proteins across diverse taxa appear to selectively utilize Mn^2+^ cations but have “tunable” selectivities toward adenine versus guanine-based alarmone substrates.

Tde-SAH contains the HD1 to HD6 motifs identified in diverse SAH, SpoT, and bifunctional long-RSH homologues (Fig. 6B) (27). The strictly conserved HD diad (His73 and Asp74) are predicted to play a key role in binding the catalytic Mn^2+^ ion within the Tde-SAH active site (20, 24, 38, 39). Mutation of Asp74 to alanine abrogated the alarmone-hydrolyzing activities of Tde-SAH (Fig. 7E), putatively by disrupting Mn^2+^ cofactor binding. This is consistent with results from the *in vivo* growth rate assays, in which the E. coli Δ*relA*Δ*spoT* (CF1693) strain complemented with Tde-SAS, and Tde-SAH_D74A_ grew considerably faster than the strain complemented with Tde-SAS and Tde-SAH. This is putatively due to the inability of the Tde-SAH_D74A_ mutant to hydrolyze the (p)ppGpp synthesized by Tde-SAS in the cell. We have elucidated the X-ray crystal structure of the Tde-SAH protein, and hence a more detailed mechanistic description of this protein will be provided elsewhere (M. Wang and R.M. Watt, unpublished data).

Based on our results, we tentatively speculate that Tde-SAS and Tde-SAH modulate alarmone (and [p]ppApp) levels in T. denticola cells via the following mechanism. In its default conformation, Tde-SAS maintains a basal level of (pp)pGpp (and [p]ppApp) synthesis. While Tde-SAS synthesizes ppGpp most efficiently, the precise ratios of pppGpp/ppGpp/pGpp produced would also be governed by the respective intracellular levels of GTP/GDP/GMP. The alarmones (and [p]ppApp) produced would be rapidly hydrolyzed by Tde-SAH due to its efficient catalytic activities. We further speculate that the TPR domain of Tde-SAS forms binding associations with components of the transcription or translational machinery that are indicative of nutrient starvation or certain extracellular stresses. These protein-binding events alter the conformation of the Tde-SAS catalytic domain, derepressing alarmone production (akin to Tde-SAS_1–246_). Upon cessation of binding to the TPR domain, the catalytic domain returns to its default conformation, and alarmone production drops to basal levels. Further investigations are required to validate this proposed mechanism.

In brief conclusion, we have established the activities of two previously undescribed lineages of SAS and SAH protein to significantly enhance our molecular understanding of (pp)pGpp and (p)ppApp metabolism within bacterial systems. Taken together, our *in vitro* and *in vivo* data suggest that the activities of Tde-SAS and Tde-SAH may be sufficient for the metabolism of (pp)pGpp in the periodontal pathogen T. denticola.

## MATERIALS AND METHODS

### Plasmid construction.

The Tde-SAS (TDE_RS08190, formerly TDE1711) and Tde-SAH (TDE_RS08100, formerly TDE1690) genes were PCR amplified from T. denticola ATCC 35405^T^ genomic DNA and cloned into pET28a (+) (Novagen, Merck Millipore) via BamHI/XhoI to create plasmids pET28a-Tde-SAS and pET28a-Tde-SAH, respectively (Table S1). The catalytic domain (Tde-SAS_1–246_) of Tde-SAS was analogously cloned to create plasmid pET28a-Tde-SAS_1–246_. The FN0926 gene from Fusobacterium nucleatum ATCC 25586^T^ was analogously cloned into pET28a to create plasmid pET28-Fn-SAS. The Tde-SAH_D74A_ mutant was created using the Phusion site-directed mutagenesis kit (Thermo Fisher Scientific, USA). The Tde-SAH and Tde-SAH_D74A_ genes were respectively subcloned into pGEX-4T1 expression vectors via BamHI/XhoI to create plasmids pGEX-Tde-SAH and pGEX-Tde-SAH_D74A_. The genes were analogously PCR amplified and cloned into pBAD33 plasmids (67). PCR primer details are shown in Table S2. Plasmid integrity was confirmed by Sanger sequencing. The plasmids were routinely maintained in E. coli DH10B (Invitrogen) and cultivated in Luria Bertani (LB) or LB-agar media (USB) containing kanamycin (50 μg/mL).

### Protein expression and purification.

Proteins were expressed from the respective recombinant plasmids that had been established in E. coli BL21(DE3) (Invitrogen), as previously described (40, 86). Briefly, 5-mL overnight cultures inoculated from single colonies were expanded into 500 mL of Terrific Broth (TB) containing kanamycin (50 μg/mL) and grown aerobically with shaking at 37°C. Protein expression was induced at OD_600_ 0.6 to 0.8 by the addition of 0.2 mM IPTG (GE Healthcare), and incubation was maintained at 25°C for 12 h. The cells were chilled on ice and collected by centrifugation (6,000 × *g*, 4°C,10 min), the supernatant was discarded, and cell pellets were washed with cold phosphate-buffered saline (PBS; pH 7.4, 25 mL) and then stored at −70°C for future use or used directly.

The cell pellets were resuspended in 15 mL ice-cold nickel-binding buffer (25 mM Tris-HCl, pH 7.4, 500 mM NaCl, 20 mM imidazole) containing protease inhibitors (cOmplete ULTRA tablet, EDTA-free, Merck Millipore) in a 50-mL Falcon tube (Corning) and lysed by sonication with ice cooling (Vibra Cell, Sonics & Materials Inc.; 36% amplitude, 2 s on, 8 s off, 10 min total). The lysates were centrifuged (16,000 × *g*, 4°C, 60 min), and the decanted supernatants were filtered (0.45-μm pore size nylon membrane syringe filter, Pall Corporation) for immediate purification by immobilized metal affinity chromatography (IMAC), on pre-equilibrated 5 mL HiTrap chelating HP columns (GE Healthcare) impregnated with nickel ions, using an AKTA purifier system (GE Healthcare), with 1 mL/min flow rate and monitoring eluent at 280 nm. After loading the filtered supernatant, the columns were washed with 5 column volumes (CVs) of nickel-binding buffer. The recombinant proteins were eluted by linear gradient elution of nickel-binding and nickel-elution buffer (25 mM Tris-HCl, pH 7.4, 500 mM NaCl, 250 mM imidazole) over 20 CV. The fractions were routinely analyzed by SDS-PAGE on 12% acrylamide/bis-acrylamide gels (37.5:1; Bio-Rad).

### Bioinformatic methods.

The DNA and protein sequences were download from the NCBI and routinely manipulated using BioEdit version 7.2.0 (87). Amino acid multiple sequence alignments (.fas files) were constructed using the ClustalW program within BioEdit, visualizing the results in EsPript 3.0 (88). TPRpred in the MPI Bioinformatics Toolkit (89) was used to identify the TPR motif locations using default parameters. TPR structure modeling was performed using the SWISS-MODEL webserver using Tde-SAS residues 256 to 410 as the input sequence with default settings (90).

Representative SAS/SAH homologues were identified using the NCBI Basic Local Alignment Search Tool (BLAST) (91), searching the NCBI GenBank nonredundant (nr) amino acid sequences and PDB sequences (www.rcsb.org) (92), and were classified into families based on previously published studies (16, 31). Phylogenetic relationships between protein amino acid sequences were inferred using GARLI v.2.0 using the default maximum likelihood (ML) approach (93). Constructed ML phylograms were visualized and edited using ITOL (94) to highlight branches from distinct SAS/SAH protein families. Details of the SAS and SAH protein sequences used for phylogenetic tree construction are summarized in Supplemental File 2.

### Enzymatic preparation of (pp)pGpp and (p)ppApp.

Nucleotides were purchased from Sigma and were of the highest purity available. The pppGpp, ppGpp, and pGpp alarmones were prepared enzymatically using the recombinant E. faecalis RelQ (EF-RelQ) protein and were purified by ion-exchange chromatography (1-mL Resource Q anion-exchange columns; GE Healthcare, USA) followed by desalting (5-mL HiTrap desalting columns; GE Healthcare, USA) as previously described (40, 86). The pppApp and ppApp nucleotides were synthesized using analogous enzymatic approaches using the recombinant SAS protein (FN0926) from F. nucleatum ATCC 25586^T^. Briefly, reaction mixtures contained 50 mM Bis-Tris propane, pH 6.8, 150 mM NaCl, 20 mM MgCl_2_, 1 mM dithiothreitol (DTT), 10 mM ATP + 10 mM ADP (to make ppApp), or 20 mM ATP (to make pppApp) and were incubated overnight at 37°C. The (p)ppApp products were purified analogously to (pp)pGpp. The concentrations of purified (pp)pGpp and (p)ppApp product solutions were determined via UV spectroscopy (Nanodrop 2000c spectrometer) based on the respective molar extinction coefficients for GTP (13,700 L.mol^−1^ · cm^−1^ at 252 nm) or ATP (15,400 L.mol^−1 ^· cm^−1^ at 259 nm), using a dilution series, as has previously been reported (40, 86).

### Nucleotide product analysis and quantification.

Enzymatic product mixtures were analyzed by anion-exchange chromatography (1 mL MonoQ 5/50 GL; GE Healthcare) using an AKTA purifier system using the following program: 100% buffer A (25 mM Tris-HCl, pH 8.0, 25 mM NaCl) for 3 CV, a linear gradient of 100% buffer A increasing to 44% buffer B (25 mM Tris-HCl, pH 8.0, 1 M NaCl) over 13 CV and then 100% buffer B for 3CV, at a flow rate of 2 mL/min. The UV absorption of the eluent was monitored at 254 nm. Nucleotides and alarmone products were identified based on their unique elution volumes, in comparison with reference standards, as previously described (40). Reference chromatograms of AMP, ADP, ATP, GMP, GDP, GTP, pGpp, ppGpp, pppGpp, ppApp, and pppApp standards run under identical conditions are shown in Fig. S1. The respective levels of AMP biproduct formed (which are equimolar to those of the corresponding enzymatic (pp)pGpp or (p)ppApp products) were quantified by measuring the respective peak areas on the chromatograms, which were then compared with a standard curve prepared from a set of AMP solutions of known concentrations (0 to 1,000 μM). The unit of activity was defined as the number of micromoles of AMP (or alarmone) synthesized per minute per micromole of protein (μmol · min^−1^ · μmol^−1^ protein). All reactions were performed in triplicate. The *y* axes of the chromatograms shown in the figures (plotted in units of milli-absorbance units, mAU) were routinely removed for the sake of clarity.

### Qualitative assays for determining (pp)pGpp/(p)ppApp/ppIpp synthesis activities.

Reaction mixtures (20 μL) containing 50 mM Tris-HCl, pH 8.8 (optimal pH), 150 mM NaCl, 10 mM MgCl_2_, 1 mM DTT, 5 mM ATP, 5 mM GMP/GDP/GTP/ADP/ATP/IDP (as indicated), and 250 nM protein were incubated at 37°C for 2 h, before being quenched by the addition of 2 mM EDTA and then snap-frozen in liquid nitrogen for future analysis. Each product mixture (20 μL) was diluted to 200 μL with Milli-Q water and analyzed by anion-exchange chromatography on a Mono Q column 5/50 GL (1 mL) as described above. All reactions were performed in triplicate.

### Kinetic analysis of alarmone synthesis by Tde-SAS and Tde-SAS_1–246_.

Kinetic parameters (*V*_max_, *k*_cat_, and *K_m_*) were calculated using the Michaelis-Menten model, incorporating results from sets of assays performed in triplicate. The assays were performed at the pH value optimal for the Tde-SAS protein (Fig. S3). Reaction mixtures (20 μL) contained 50 mM Tris-HCl, pH 8.8, 150 mM NaCl, 10 mM MgCl_2_, 1 mM DTT, 5 mM ATP, 0 to 10 mM GMP/GDP/GTP, 250 nM protein (Tde-SAS/Tde-SAS_1–246_), with or without 100 μM pppGpp/ppGpp (as a putative allosteric modulator). Alarmone synthesis rates were determined by quantifying AMP levels as described above.

### Quantitative determination of Tde-SAH (pp)pGpp and (p)ppApp rates of hydrolysis.

The respective rates of pppGpp, ppGpp, pGpp, pppApp, and ppApp hydrolysis by Tde-SAH were determined using an enzyme-coupled continuous fluorescent assay (EnzChek phosphate assay kit; Thermo Fisher Scientific, USA) as previously described (40). Assays were performed at the pH value optimal for the Tde-SAH protein (Fig. S3). Briefly, reaction mixtures in 96-well plates (200 μL) contained 50 mM Tris-HCl, pH 8.4, 150 mM NaCl, 1 mM MnCl_2_, 1 mM DTT, 200 μM 2-amino-6-mercapto-7-methylpurine ribonucleoside (MESG), 0.2 units PNP, 3 μM recombinant S. aureus inorganic pyrophosphatase protein (Sa-PpaC, SAV1919), and 250 nM Tde-SAH protein. The assay mixtures were preincubated at 37°C for 10 min, and then the assays were initiated by the addition of the respective pppGpp, ppGpp, or pGpp substrate (0 to 500 μM; for assays used for Michaelis-Menton kinetic parameter determination) or 200 μM pppGpp, ppGpp, pGpp, pppApp, or ppApp (for assays used to determine specific rate of hydrolysis). Reaction mixtures were incubated at 37°C for 15 min with OD_360_ readings taken every 30 s, using a SpectraMax M2e multilabel microplate reader (Molecular Devices). The rate of hydrolysis was normalized using a phosphate standard curve constructed according to the manufacturer’s recommended protocol.

### Evaluation of *in vivo* (p)ppGpp production using E. coli Δ*relA*Δ*spoT* reporter system.

Cellular (p)ppGpp levels were evaluated using growth assays with the E. coli Δ*relA*Δ*spoT* (CF1693) strain (from Michael Cashel) (23), which was cultivated in MOPS minimal medium (1× MOPS mixture, 1.32 mM K_2_HPO_4_, 1% glucose as carbon source) as previously described (31, 36) with minor modifications. Plasmids and strains used are listed in Table S1. pBAD33 plasmids containing the Tde-SAS, Tde-SAS_1–246_, S. aureus RelP (Sa-RelP, NWMN_2405), or Tde-SAH gene or no insert (pBAD33-empty, negative control) were transformed into E. coli CF1693 and were propagated at 37°C in LB medium containing chloramphenicol (Cm, 25 μg/mL) to ensure plasmid maintenance. Wild-type E. coli MG1655 (CF1648) (23) transformed with pBAD33 was included as an additional control. Aliquots (10 μL) of overnight cultures (single colonies inoculated into 5 mL of LB + Cm, incubated at 37°C for 16 h) were diluted to a final OD_600_ of 0.08 into MOPS minimal medium (990 μL) containing chloramphenicol (25 μg/mL) and 0.2% arabinose to induce protein expression. Aliquots (200 μL) were pipetted into 96-well plates, which were incubated at 37°C for 10 h with OD_600_ readings taken every 10 min (immediately after a few seconds of automated agitation), using a SpectraMax M2e multilabel microplate reader (Molecular Devices).

Analogous sets of growth assays were performed in E. coli CF1693 transformed with pairs of pBAD33 and pGEX-4T1 plasmids respectively containing Tde-SAS, Tde-SAH, Tde-SAH_D74A_ (hydrolytically inactive), or no genetic insert (negative control). Transformed E. coli CF1693 strains were routinely propagated at 37°C in LB medium containing ampicillin (100 μg/mL) and chloramphenicol (25 μg/mL) for stable plasmid maintenance. These strains were used in growth assays performed analogously to those described above, except using MOPS minimal medium containing ampicillin (100 μg/mL), chloramphenicol (25 μg/mL), arabinose (0.2%), and IPTG (0.2 mM) to induce protein expression. Throughout these experiments, we were fastidious with the cultivation and restreaking of this “(p)ppGpp zero” strain to reduce the likelihood of spontaneous suppressor (e.g., RNA polymerase) mutations occurring (66). Additional experimental details are included in the Supplemental Methods.

### Data availability.

All research materials and primary data sets are available upon request (Rory M. Watt, rmwatt@hku.hk).

## References

[1] Anderson BW, Fung DK, Wang JD. 2021. Regulatory themes and variations by the stress-signaling nucleotide alarmones (p)ppGpp in bacteria. Annu Rev Genet 55:115–133. doi:10.1146/annurev-genet-021821-025827. 34416118 PMC12582393

[2] Bange G, Brodersen DE, Liuzzi A, Steinchen W. 2021. Two P or not two P: understanding regulation by the bacterial second messengers (p)ppGpp. Annu Rev Microbiol 75:383–406. doi:10.1146/annurev-micro-042621-122343. 34343020

[3] Irving SE, Choudhury NR, Corrigan RM. 2021. The stringent response and physiological roles of (pp)pGpp in bacteria. Nat Rev Microbiol 19:256–271. doi:10.1038/s41579-020-00470-y. 33149273

[4] Kundra S, Colomer-Winter C, Lemos JA. 2020. Survival of the fittest: the relationship of (p)ppGpp with bacterial virulence. Front Microbiol 11:601417. doi:10.3389/fmicb.2020.601417. 33343543 PMC7744563

[5] Steinchen W, Zegarra V, Bange G. 2020. (p)ppGpp: magic modulators of bacterial physiology and metabolism. Front Microbiol 11:2072. doi:10.3389/fmicb.2020.02072. 33013756 PMC7504894

[6] Hauryliuk V, Atkinson GC, Murakami KS, Tenson T, Gerdes K. 2015. Recent functional insights into the role of (p)ppGpp in bacterial physiology. Nat Rev Microbiol 13:298–309. doi:10.1038/nrmicro3448. 25853779 PMC4659695

[7] Dalebroux ZD, Svensson SL, Gaynor EC, Swanson MS. 2010. ppGpp conjures bacterial virulence. Microbiol Mol Biol Rev 74:171–199. doi:10.1128/MMBR.00046-09. 20508246 PMC2884408

[8] Potrykus K, Cashel M. 2008. (p)ppGpp: still magical? Annu Rev Microbiol 62:35–51. doi:10.1146/annurev.micro.62.081307.162903. 18454629

[9] Cashel M, Gallant J. 1969. Two compounds implicated in the function of the RC gene of *Escherichia coli*. Nature 221:838–841. doi:10.1038/221838a0. 4885263

[10] Haseltine WA, Block R. 1973. Synthesis of guanosine tetra- and pentaphosphate requires the presence of a codon-specific, uncharged transfer ribonucleic acid in the acceptor site of ribosomes. Proc Natl Acad Sci USA 70:1564–1568. doi:10.1073/pnas.70.5.1564. 4576025 PMC433543

[11] Kriel A, Bittner AN, Kim SH, Liu K, Tehranchi AK, Zou WY, Rendon S, Chen R, Tu BP, Wang JD. 2012. Direct regulation of GTP homeostasis by (p)ppGpp: a critical component of viability and stress resistance. Mol Cell 48:231–241. doi:10.1016/j.molcel.2012.08.009. 22981860 PMC3483369

[12] Kuroda A, Murphy H, Cashel M, Kornberg A. 1997. Guanosine tetra- and pentaphosphate promote accumulation of inorganic polyphosphate in *Escherichia coli*. J Biol Chem 272:21240–21243. doi:10.1074/jbc.272.34.21240. 9261133

[13] Varik V, Oliveira SRA, Hauryliuk V, Tenson T. 2017. HPLC-based quantification of bacterial housekeeping nucleotides and alarmone messengers ppGpp and pppGpp. Sci Rep 7:11022. doi:10.1038/s41598-017-10988-6. 28887466 PMC5591245

[14] Gaca AO, Kudrin P, Colomer-Winter C, Beljantseva J, Liu K, Anderson B, Wang JD, Rejman D, Potrykus K, Cashel M, Hauryliuk V, Lemos JA. 2015. From (p)ppGpp to (pp)pGpp: characterization of regulatory effects of pGpp synthesized by the small alarmone synthetase of *Enterococcus faecalis*. J Bacteriol 197:2908–2919. doi:10.1128/JB.00324-15. 26124242 PMC4542164

[15] Yang J, Anderson BW, Turdiev A, Turdiev H, Stevenson DM, Amador-Noguez D, Lee VT, Wang JD. 2020. The nucleotide pGpp acts as a third alarmone in *Bacillus*, with functions distinct from those of (p)ppGpp. Nat Commun 11:5388. doi:10.1038/s41467-020-19166-1. 33097692 PMC7584652

[16] Atkinson GC, Tenson T, Hauryliuk V. 2011. The RelA/SpoT homolog (RSH) superfamily: distribution and functional evolution of ppGpp synthetases and hydrolases across the tree of life. PLoS One 6:e23479. doi:10.1371/journal.pone.0023479. 21858139 PMC3153485

[17] Mittenhuber G. 2001. Comparative genomics and evolution of genes encoding bacterial (p)ppGpp synthetases/hydrolases (the Rel, RelA and SpoT proteins). J Mol Microbiol Biotechnol 3:585–600. 11545276

[18] Mechold U, Murphy H, Brown L, Cashel M. 2002. Intramolecular regulation of the opposing (p)ppGpp catalytic activities of Rel(Seq), the Rel/Spo enzyme from *Streptococcus equisimilis*. J Bacteriol 184:2878–2888. doi:10.1128/JB.184.11.2878-2888.2002. 12003927 PMC135074

[19] Avarbock D, Avarbock A, Rubin H. 2000. Differential regulation of opposing RelMtb activities by the aminoacylation state of a tRNA·ribosome·mRNA·RelMtb complex. Biochemistry 39:11640–11648. doi:10.1021/bi001256k. 10995231

[20] Aravind L, Koonin EV. 1998. The HD domain defines a new superfamily of metal-dependent phosphohydrolases. Trends Biochem Sci 23:469–472. doi:10.1016/s0968-0004(98)01293-6. 9868367

[21] Sy J. 1977. *In vitro* degradation of guanosine 5'-diphosphate, 3'-diphosphate. Proc Natl Acad Sci USA 74:5529–5533. doi:10.1073/pnas.74.12.5529. 414222 PMC431794

[22] Sy J, Lipmann F. 1973. Identification of the synthesis of guanosine tetraphosphate (MS I) as insertion of a pyrophosphoryl group into the 3'-position in guanosine 5'-diphosphate. Proc Natl Acad Sci USA 70:306–309. doi:10.1073/pnas.70.2.306. 4346881 PMC433245

[23] Xiao H, Kalman M, Ikehara K, Zemel S, Glaser G, Cashel M. 1991. Residual guanosine 3',5'-bispyrophosphate synthetic activity of relA null mutants can be eliminated by spoT null mutations. J Biol Chem 266:5980–5990. doi:10.1016/S0021-9258(19)67694-5. 2005134

[24] Hogg T, Mechold U, Malke H, Cashel M, Hilgenfeld R. 2004. Conformational antagonism between opposing active sites in a bifunctional RelA/SpoT homolog modulates (p)ppGpp metabolism during the stringent response. Cell 117:57–68. doi:10.1016/s0092-8674(04)00260-0. 15066282

[25] Avarbock A, Avarbock D, Teh JS, Buckstein M, Wang ZM, Rubin H. 2005. Functional regulation of the opposing (p)ppGpp synthetase/hydrolase activities of RelMtb from *Mycobacterium tuberculosis*. Biochemistry 44:9913–9923. doi:10.1021/bi0505316. 16026164

[26] Ronneau S, Hallez R. 2019. Make and break the alarmone: regulation of (p)ppGpp synthetase/hydrolase enzymes in bacteria. FEMS Microbiol Rev 43:389–400. doi:10.1093/femsre/fuz009. 30980074 PMC6606846

[27] Steinchen W, Bange G. 2016. The magic dance of the alarmones (p)ppGpp. Mol Microbiol 101:531–544. doi:10.1111/mmi.13412. 27149325

[28] Lemos JA, Lin VK, Nascimento MM, Abranches J, Burne RA. 2007. Three gene products govern (p)ppGpp production by *Streptococcus mutans*. Mol Microbiol 65:1568–1581. doi:10.1111/j.1365-2958.2007.05897.x. 17714452

[29] Nanamiya H, Kasai K, Nozawa A, Yun CS, Narisawa T, Murakami K, Natori Y, Kawamura F, Tozawa Y. 2008. Identification and functional analysis of novel (p)ppGpp synthetase genes in *Bacillus subtilis*. Mol Microbiol 67:291–304. doi:10.1111/j.1365-2958.2007.06018.x. 18067544

[30] Das B, Pal RR, Bag S, Bhadra RK. 2009. Stringent response in *Vibrio cholerae*: genetic analysis of spoT gene function and identification of a novel (p)ppGpp synthetase gene. Mol Microbiol 72:380–398. doi:10.1111/j.1365-2958.2009.06653.x. 19298370

[31] Jimmy S, Saha CK, Kurata T, Stavropoulos C, Oliveira SRA, Koh A, Cepauskas A, Takada H, Rejman D, Tenson T, Strahl H, Garcia-Pino A, Hauryliuk V, Atkinson GC. 2020. A widespread toxin-antitoxin system exploiting growth control via alarmone signaling. Proc Natl Acad Sci USA 117:10500–10510. doi:10.1073/pnas.1916617117. 32345719 PMC7229694

[32] Geiger T, Kastle B, Gratani FL, Goerke C, Wolz C. 2014. Two small (p)ppGpp synthases in *Staphylococcus aureus* mediate tolerance against cell envelope stress conditions. J Bacteriol 196:894–902. doi:10.1128/JB.01201-13. 24336937 PMC3911181

[33] Abranches J, Martinez AR, Kajfasz JK, Chavez V, Garsin DA, Lemos JA. 2009. The molecular alarmone (p)ppGpp mediates stress responses, vancomycin tolerance, and virulence in *Enterococcus faecalis*. J Bacteriol 191:2248–2256. doi:10.1128/JB.01726-08. 19168608 PMC2655485

[34] Gaca AO, Kajfasz JK, Miller JH, Liu K, Wang JD, Abranches J, Lemos JA. 2013. Basal levels of (p)ppGpp in *Enterococcus faecalis*: the magic beyond the stringent response. mBio 4:e00646-13. doi:10.1128/mBio.00646-13. 24065631 PMC3781836

[35] Poudel A, Pokhrel A, Oludiran A, Coronado EJ, Alleyne K, Gilfus MM, Gurung RK, Adhikari SB, Purcell EB. 2022. Unique features of alarmone metabolism in *Clostridioides difficile*. J Bacteriol 204:e00575-21. doi:10.1128/jb.00575-21. 35254095 PMC9017329

[36] Ruwe M, Kalinowski J, Persicke M. 2017. Identification and functional characterization of small alarmone synthetases in *Corynebacterium glutamicum*. Front Microbiol 8:1601. doi:10.3389/fmicb.2017.01601. 28871248 PMC5566576

[37] Ruwe M, Ruckert C, Kalinowski J, Persicke M. 2018. Functional characterization of a small alarmone hydrolase in *Corynebacterium glutamicum*. Front Microbiol 9:916. doi:10.3389/fmicb.2018.00916. 29867827 PMC5954133

[38] Steinchen W, Ahmad S, Valentini M, Eilers K, Majkini M, Altegoer F, Lechner M, Filloux A, Whitney JC, Bange G. 2021. Dual role of a (p)ppGpp- and (p)ppApp-degrading enzyme in biofilm formation and interbacterial antagonism. Mol Microbiol 115:1339–1356. doi:10.1111/mmi.14684. 33448498

[39] Bisiak F, Chrenkova A, Zhang SD, Pedersen JN, Otzen DE, Zhang YE, Brodersen DE. 2022. Structural variations between small alarmone hydrolase dimers support different modes of regulation of the stringent response. J Biol Chem 298:102–142. 10.1016/j.jbc.2022.102142 PMC9293644 35714769

[40] Yang N, Xie S, Tang NY, Choi MY, Wang Y, Watt RM. 2019. The Ps and Qs of alarmone synthesis in *Staphylococcus aureus*. PLoS One 14:e0213630. doi:10.1371/journal.pone.0213630. 31613897 PMC6793942

[41] Steinchen W, Vogt MS, Altegoer F, Giammarinaro PI, Horvatek P, Wolz C, Bange G. 2018. Structural and mechanistic divergence of the small (p)ppGpp synthetases RelP and RelQ. Sci Rep 8:2195. doi:10.1038/s41598-018-20634-4. 29391580 PMC5794853

[42] Manav MC, Beljantseva J, Bojer MS, Tenson T, Ingmer H, Hauryliuk V, Brodersen DE. 2018. Structural basis for (p)ppGpp synthesis by the *Staphylococcus aureus* small alarmone synthetase RelP. J Biol Chem 293:3254–3264. doi:10.1074/jbc.RA117.001374. 29326162 PMC5836137

[43] Dasgupta S, Basu P, Pal RR, Bag S, Bhadra RK. 2014. Genetic and mutational characterization of the small alarmone synthetase gene relV of *Vibrio cholerae*. Microbiology 160:1855–1866. doi:10.1099/mic.0.079319-0. 24987103

[44] Murdeshwar MS, Chatterji D. 2012. MS_RHII-RSD, a dual-function RNase HII-(p)ppGpp synthetase from *Mycobacterium smegmatis*. J Bacteriol 194:4003–4014. doi:10.1128/JB.00258-12. 22636779 PMC3416543

[45] Petchiappan A, Naik SY, Chatterji D. 2020. RelZ-mediated stress response in *Mycobacterium smegmatis*: pGpp synthesis and its regulation. J Bacteriol 202:e004444-19. doi:10.1128/JB.00444-19. PMC6941529 31659009

[46] Fung DK, Yang J, Stevenson DM, Amador-Noguez D, Wang JD. 2020. Small alarmone aynthetase SasA expression leads to concomitant accumulation of pGpp, ppApp, and AppppA in *Bacillus subtilis*. Front Microbiol 11:2083. doi:10.3389/fmicb.2020.02083. 32983059 PMC7492591

[47] Ahmad S, Wang B, Walker MD, Tran HR, Stogios PJ, Savchenko A, Grant RA, McArthur AG, Laub MT, Whitney JC. 2019. An interbacterial toxin inhibits target cell growth by synthesizing (p)ppApp. Nature 575:674–678. doi:10.1038/s41586-019-1735-9. 31695193 PMC6883173

[48] Kurata T, Brodiazhenko T, Oliveira SRA, Roghanian M, Sakaguchi Y, Turnbull KJ, Bulvas O, Takada H, Tamman H, Ainelo A, Pohl R, Rejman D, Tenson T, Suzuki T, Garcia-Pino A, Atkinson GC, Hauryliuk V. 2021. RelA-SpoT Homolog toxins pyrophosphorylate the CCA end of tRNA to inhibit protein synthesis. Mol Cell 81:3160–3170.e9. doi:10.1016/j.molcel.2021.06.005. 34174184

[49] Sobala M, Bruhn-Olszewska B, Cashel M, Potrykus K. 2019. *Methylobacterium extorquens* RSH enzyme synthesizes (p)ppGpp and pppApp *in vitro* and *in vivo*, and leads to discovery of pppApp synthesis in *Escherichia coli*. Front Microbiol 10:859. doi:10.3389/fmicb.2019.00859. 31068922 PMC6491832

[50] Potrykus K, Thomas NE, Bruhn-Olszewska B, Sobala M, Dylewski M, James T, Cashel M. 2020. Estimates of RelSeq, Mesh1, and SAHMex hydrolysis of (p)ppGpp and (p)ppApp by thin layer chromatography and NADP/NADH coupled assays. Front Microbiol 11:581271. doi:10.3389/fmicb.2020.581271. 33193211 PMC7644958

[51] Dashper SG, Seers CA, Tan KH, Reynolds EC. 2011. Virulence factors of the oral spirochete *Treponema denticola*. J Dent Res 90:691–703. doi:10.1177/0022034510385242. 20940357 PMC3144123

[52] Visser MB, Ellen RP. 2011. New insights into the emerging role of oral spirochaetes in periodontal disease. Clin Microbiol Infect 17:502–512. doi:10.1111/j.1469-0691.2011.03460.x. 21414084

[53] Kinane DF, Stathopoulou PG, Papapanou PN. 2017. Periodontal diseases. Nat Rev Dis Primers 3:17038. doi:10.1038/nrdp.2017.38. 28805207

[54] Kononen E, Gursoy M, Gursoy UK. 2019. Periodontitis: a multifaceted disease of tooth-supporting tissues. J Clin Med 8:1135. doi:10.3390/jcm8081135. 31370168 PMC6723779

[55] Ishihara K. 2010. Virulence factors of *Treponema denticola*. Periodontol 2000 54:117–135. doi:10.1111/j.1600-0757.2009.00345.x. 20712637

[56] Tonetti MS, Jepsen S, Jin L, Otomo-Corgel J. 2017. Impact of the global burden of periodontal diseases on health, nutrition and wellbeing of mankind: a call for global action. J Clin Periodontol 44:456–462. doi:10.1111/jcpe.12732. 28419559

[57] You M, Mo SS, Leung WK, Watt RM. 2013. Comparative analysis of oral treponemes associated with periodontal health and disease. BMC Infect Dis 13:174. doi:10.1186/1471-2334-13-174. 23578286 PMC3637317

[58] Dewhirst FE, Chen T, Izard J, Paster BJ, Tanner AC, Yu WH, Lakshmanan A, Wade WG. 2010. The human oral microbiome. J Bacteriol 192:5002–5017. doi:10.1128/JB.00542-10. 20656903 PMC2944498

[59] Seshadri R, Myers GSA, Tettelin H, Eisen JA, Heidelberg JF, Dodson RJ, Davidsen TM, DeBoy RT, Fouts DE, Haft DH, Selengut J, Ren Q, Brinkac LM, Madupu R, Kolonay J, Durkin SA, Daugherty SC, Shetty J, Shvartsbeyn A, Gebregeorgis E, Geer K, Tsegaye G, Malek J, Ayodeji B, Shatsman S, McLeod MP, Smajs D, Howell JK, Pal S, Amin A, Vashisth P, McNeill TZ, Xiang Q, Sodergren E, Baca E, Weinstock GM, Norris SJ, Fraser CM, Paulsen IT. 2004. Comparison of the genome of the oral pathogen *Treponema denticola* with other spirochete genomes. Proc Natl Acad Sci USA 101:5646–5651. doi:10.1073/pnas.0307639101. 15064399 PMC397461

[60] D'Andrea LD, Regan L. 2003. TPR proteins: the versatile helix. Trends Biochem Sci 28:655–662. doi:10.1016/j.tibs.2003.10.007. 14659697

[61] Karpenahalli MR, Lupas AN, Soding J. 2007. TPRpred: a tool for prediction of TPR-, PPR- and SEL1-like repeats from protein sequences. BMC Bioinformatics 8:2. doi:10.1186/1471-2105-8-2. 17199898 PMC1774580

[62] Turnbull KJ, Dzhygyr I, Lindemose S, Hauryliuk V, Roghanian M. 2019. Intramolecular interactions dominate the autoregulation of *Escherichia coli* stringent factor RelA. Front Microbiol 10:1966. doi:10.3389/fmicb.2019.01966. 31507571 PMC6719525

[63] Fontana A, de Laureto PP, Spolaore B, Frare E, Picotti P, Zambonin M. 2004. Probing protein structure by limited proteolysis. Acta Biochim Pol 51:299–321. 15218531

[64] Steinchen W, Schuhmacher JS, Altegoer F, Fage CD, Srinivasan V, Linne U, Marahiel MA, Bange G. 2015. Catalytic mechanism and allosteric regulation of an oligomeric (p)ppGpp synthetase by an alarmone. Proc Natl Acad Sci USA 112:13348–13353. doi:10.1073/pnas.1505271112. 26460002 PMC4629338

[65] Sun D, Lee G, Lee JH, Kim HY, Rhee HW, Park SY, Kim KJ, Kim Y, Kim BY, Hong JI, Park C, Choy HE, Kim JH, Jeon YH, Chung J. 2010. A metazoan ortholog of SpoT hydrolyzes ppGpp and functions in starvation responses. Nat Struct Mol Biol 17:1188–1194. doi:10.1038/nsmb.1906. 20818390

[66] Murphy H, Cashel M. 2003. Isolation of RNA polymerase suppressors of a (p)ppGpp deficiency. Methods Enzymol 371:596–601. doi:10.1016/S0076-6879(03)71044-1. 14712731

[67] Guzman LM, Belin D, Carson MJ, Beckwith J. 1995. Tight regulation, modulation, and high-level expression by vectors containing the arabinose PBAD promoter. J Bacteriol 177:4121–4130. doi:10.1128/jb.177.14.4121-4130.1995. 7608087 PMC177145

[68] Lacap-Bugler DC, Jiang J, Huo YB, Chan Y, Leung FC, Watt RM. 2014. Complete genome sequence of the oral spirochete bacterium *Treponema putidum* strain OMZ 758^T^ (ATCC 700334^T^). Genome Announc 2:e01076-14. doi:10.1128/genomeA.01076-14. 25342686 PMC4208330

[69] Svartstrom O, Mushtaq M, Pringle M, Segerman B. 2013. Genome-wide relatedness of *Treponema pedis*, from gingiva and necrotic skin lesions of pigs, with the human oral pathogen *Treponema denticola*. PLoS One 8:e71281. doi:10.1371/journal.pone.0071281. 23977007 PMC3747143

[70] Jaiswal AK, Tiwari S, Jamal SB, de Castro Oliveira L, Alves LG, Azevedo V, Ghosh P, Oliveira CJF, Soares SC. 2020. The pan-genome of *Treponema pallidum* reveals differences in genome plasticity between subspecies related to venereal and non-venereal syphilis. BMC Genomics 21:33. doi:10.1186/s12864-019-6430-6. 31924165 PMC6953169

[71] Beljantseva J, Kudrin P, Andresen L, Shingler V, Atkinson GC, Tenson T, Hauryliuk V. 2017. Negative allosteric regulation of *Enterococcus faecalis* small alarmone synthetase RelQ by single-stranded RNA. Proc Natl Acad Sci USA 114:3726–3731. doi:10.1073/pnas.1617868114. 28320944 PMC5389274

[72] Bennett BD, Kimball EH, Gao M, Osterhout R, Van Dien SJ, Rabinowitz JD. 2009. Absolute metabolite concentrations and implied enzyme active site occupancy in *Escherichia coli*. Nat Chem Biol 5:593–599. doi:10.1038/nchembio.186. 19561621 PMC2754216

[73] Buckstein MH, He J, Rubin H. 2008. Characterization of nucleotide pools as a function of physiological state in *Escherichia coli*. J Bacteriol 190:718–726. doi:10.1128/JB.01020-07. 17965154 PMC2223692

[74] Zbornikova E, Knejzlik Z, Hauryliuk V, Krasny L, Rejman D. 2019. Analysis of nucleotide pools in bacteria using HPLC-MS in HILIC mode. Talanta 205:120161. doi:10.1016/j.talanta.2019.120161. 31450400

[75] Zeytuni N, Zarivach R. 2012. Structural and functional discussion of the tetra-trico-peptide repeat, a protein interaction module. Structure 20:397–405. doi:10.1016/j.str.2012.01.006. 22404999

[76] Cerveny L, Straskova A, Dankova V, Hartlova A, Ceckova M, Staud F, Stulik J. 2013. Tetratricopeptide repeat motifs in the world of bacterial pathogens: role in virulence mechanisms. Infect Immun 81:629–635. doi:10.1128/IAI.01035-12. 23264049 PMC3584863

[77] Speltz EB, Nathan A, Regan L. 2015. Design of protein-peptide interaction modules for assembling supramolecular structures *in vivo* and *in vitro*. ACS Chem Biol 10:2108–2115. doi:10.1021/acschembio.5b00415. 26131725

[78] Mueller CA, Broz P, Cornelis GR. 2008. The type III secretion system tip complex and translocon. Mol Microbiol 68:1085–1095. doi:10.1111/j.1365-2958.2008.06237.x. 18430138

[79] Wang Y, Pannuri AA, Ni D, Zhou H, Cao X, Lu X, Romeo T, Huang Y. 2016. Structural basis for translocation of a biofilm-supporting exopolysaccharide across the bacterial outer membrane. J Biol Chem 291:10046–10057. doi:10.1074/jbc.M115.711762. 26957546 PMC4858958

[80] Rhaese HJ, Hoch JA, Groscurth R. 1977. Studies on the control of development: isolation of *Bacillus subtilis* mutants blocked early in sporulation and defective in synthesis of highly phosphorylated nucleotides. Proc Natl Acad Sci USA 74:1125–1129. doi:10.1073/pnas.74.3.1125. 403525 PMC430617

[81] Travers AA. 1978. ppApp alters transcriptional selectivity of *Escherichia coli* RNA polymerase. FEBS Lett 94:345–348. doi:10.1016/0014-5793(78)80973-9. 359364

[82] Bruhn-Olszewska B, Molodtsov V, Sobala M, Dylewski M, Murakami KS, Cashel M, Potrykus K. 2018. Structure-function comparisons of (p)ppApp vs (p)ppGpp for *Escherichia coli* RNA polymerase binding sites and for rrnB P1 promoter regulatory responses *in vitro*. Biochim Biophys Acta Gene Regul Mech 1861:731–742. doi:10.1016/j.bbagrm.2018.07.005. 30012465 PMC6114088

[83] Chau NYE, Ahmad S, Whitney JC, Coombes BK. 2021. Emerging and divergent roles of pyrophosphorylated nucleotides in bacterial physiology and pathogenesis. PLoS Pathog 17:e1009532. doi:10.1371/journal.ppat.1009532. 33984072 PMC8118318

[84] Ding CC, Rose J, Sun T, Wu J, Chen PH, Lin CC, Yang WH, Chen KY, Lee H, Xu E, Tian S, Akinwuntan J, Zhao J, Guan Z, Zhou P, Chi JT. 2020. MESH1 is a cytosolic NADPH phosphatase that regulates ferroptosis. Nat Metab 2:270–277. doi:10.1038/s42255-020-0181-1. 32462112 PMC7252213

[85] Mestre AA, Zhou P, Chi JT. 2022. Metazoan stringent-like response mediated by MESH1 phenotypic conservation via distinct mechanisms. Comput Struct Biotechnol J 20:2680–2684. doi:10.1016/j.csbj.2022.05.001. 35685369 PMC9166373

[86] Choi MY, Wang Y, Wong LL, Lu BT, Chen WY, Huang JD, Tanner JA, Watt RM. 2012. The two PPX-GppA homologues from *Mycobacterium tuberculosis* have distinct biochemical activities. PLoS One 7:e42561. doi:10.1371/journal.pone.0042561. 22880033 PMC3411833

[87] Hall TA. 1999. BioEdit: a user-friendly biological sequence alignment editor and analysis program for Windows 95/98/NT. Nucleic Acids Symp Ser 41:95–98.

[88] Robert X, Gouet P. 2014. Deciphering key features in protein structures with the new ENDscript server. Nucleic Acids Res 42:W320–W324. doi:10.1093/nar/gku316. 24753421 PMC4086106

[89] Zimmermann L, Stephens A, Nam SZ, Rau D, Kubler J, Lozajic M, Gabler F, Soding J, Lupas AN, Alva V. 2018. A completely reimplemented MPI bioinformatics toolkit with a new HHpred server at its core. J Mol Biol 430:2237–2243. doi:10.1016/j.jmb.2017.12.007. 29258817

[90] Biasini M, Bienert S, Waterhouse A, Arnold K, Studer G, Schmidt T, Kiefer F, Gallo Cassarino T, Bertoni M, Bordoli L, Schwede T. 2014. SWISS-MODEL: modelling protein tertiary and quaternary structure using evolutionary information. Nucleic Acids Res 42:W252–W258. doi:10.1093/nar/gku340. 24782522 PMC4086089

[91] Altschul SF, Gish W, Miller W, Myers EW, Lipman DJ. 1990. Basic local alignment search tool. J Mol Biol 215:403–410. doi:10.1016/S0022-2836(05)80360-2. 2231712

[92] Burley SK, Berman HM, Bhikadiya C, Bi C, Chen L, Di Costanzo L, Christie C, Dalenberg K, Duarte JM, Dutta S, Feng Z, Ghosh S, Goodsell DS, Green RK, Guranovic V, Guzenko D, Hudson BP, Kalro T, Liang Y, Lowe R, Namkoong H, Peisach E, Periskova I, Prlic A, Randle C, Rose A, Rose P, Sala R, Sekharan M, Shao C, Tan L, Tao YP, Valasatava Y, Voigt M, Westbrook J, Woo J, Yang H, Young J, Zhuravleva M, Zardecki C. 2019. RCSB Protein Data Bank: biological macromolecular structures enabling research and education in fundamental biology, biomedicine, biotechnology and energy. Nucleic Acids Res 47:D464–D474. doi:10.1093/nar/gky1004. 30357411 PMC6324064

[93] Zwickl DJ. 2006. Genetic algorithm approaches for the phylogenetic analysis of large biological sequence datasets under the maximum likelihood criterion. PhD thesis. University of Texas at Austin, Austin, Texas.

[94] Letunic I, Bork P. 2021. Interactive Tree Of Life (iTOL) v5: an online tool for phylogenetic tree display and annotation. Nucleic Acids Res 49:W293–W296. doi:10.1093/nar/gkab301. 33885785 PMC8265157

