# Supplementary material for: Functional Characterization of Small Alarmone Synthetase and Small Alarmone Hydrolase Proteins from Treponema denticola
Source: Microbiol Spectr. 2023 Jun 8;11(4):e05100-22. doi: 10.1128/spectrum.05100-22 (PMC10434055; doi:10.1128/spectrum.05100-22)
Supplement: Supplemental file 1 — Supplemental material. Download spectrum.05100-22-s0001.pdf, PDF file, 0.9 MB [file spectrum.05100-22-s0001.pdf]

SUPPLEMENTARY MATERIAL

Functional characterization of small alarmone synthetase and small alarmone hydrolase proteins from *Treponema denticola*

Miao Wang[1], Nga-Yeung Tang[2,3], Shujie Xie[1], Rory M. Watt[1*].

[1.]Faculty of Dentistry, The University of Hong Kong, Pok Fu Lam, Hong Kong SAR, China

[2.]Department of Pathology and Laboratory Medicine, Beaumont Health, Royal Oak, MI, USA

[3.]Department of Pathology and Laboratory Medicine, Oakland University William Beaumont School of Medicine, Auburn Hills, MI, USA

***Corresponding author:**

Rory M. Watt, Faculty of Dentistry, Prince Philip Dental Hospital, 34 Hospital Road, Sai Ying Pun, Hong Kong SAR, China

Tel: (+852) 2859 0482. Fax: (+852) 2547 6133. Email: rmwatt@hku.hk

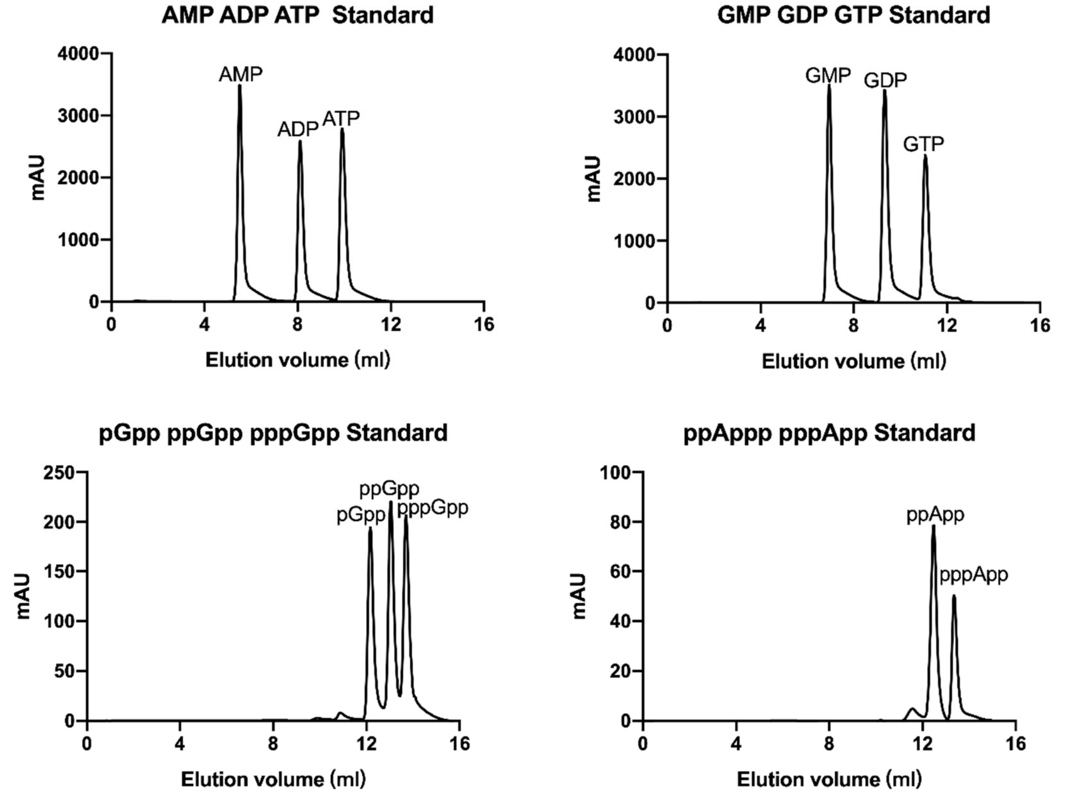

Figure S1. Reference anion exchange chromatograms of nucleotide substrates and (pp)pGpp and (p)ppApp products.
Nucleotide mixtures were analyzed on a Mono Q 5/50 GL (1 mL) column (Cytivia) using an AKTA purifier system (GE Healthcare) using a linear gradient elution program starting with 100% buffer A (25 mM Tris-HCl pH 8.0, 25 mM NaCl), increasing to 100% buffer B (25 mM Tris-HCl pH 8.0, 1 M NaCl) over 13 column volumes, followed by 100% buffer B. The UV absorption of the eluent was monitored at 254 nm and is plotted on the Y-axis in milli-absorbance units (mAU). The elution volume (in ml) is plotted on the X-axis. **Top left panel**: Mixture of AMP, ADP and ATP. **Top right panel**: Mixture of GMP, GDP and GTP. **Bottom left panel**: Mixture of pGpp, ppGpp and pppGpp. **Bottom right panel**: mixture of ppApp and pppApp.

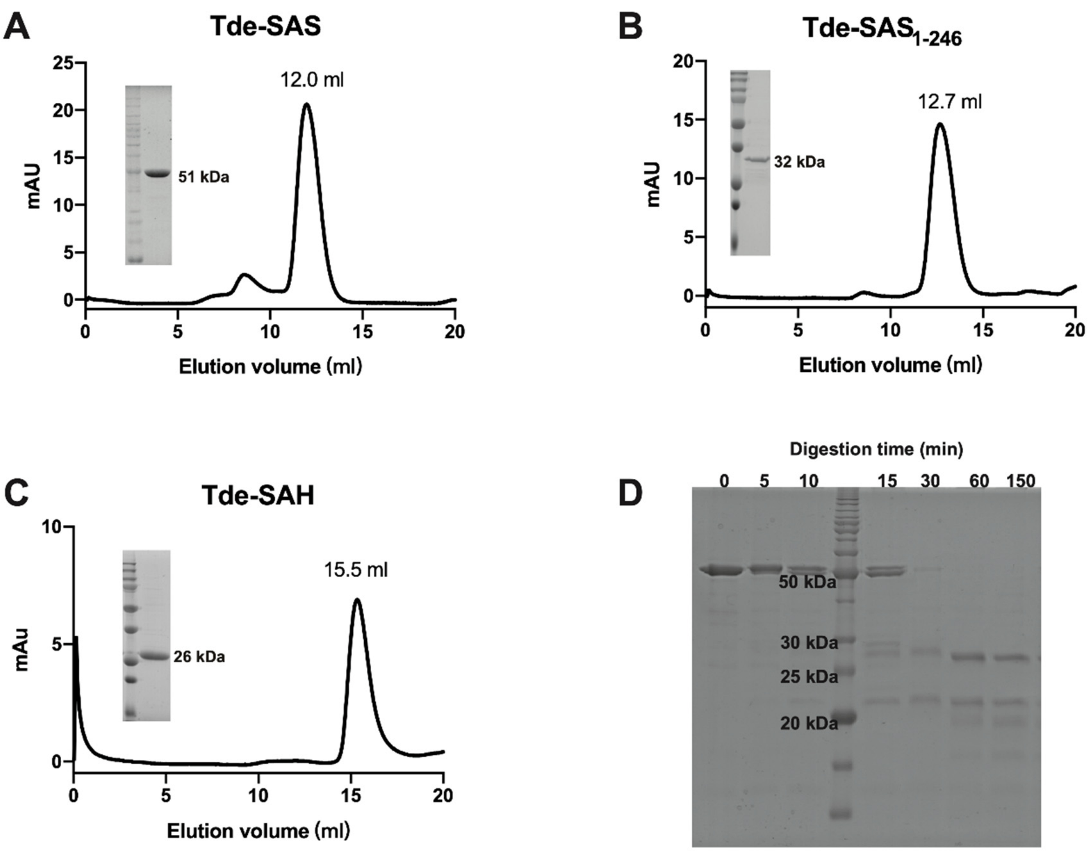

Figure S2. Size exclusion chromatography analysis of Tde-SAS, Tde-SAS and Tde-SAS$_{1\text{-}246}$; limited proteolysis of Tde-SAS

Panels A-C respectively show the size exclusion chromatograms of purified recombinant proteins used in this study. The panel insets respectively show the Coomassie brilliant blue (CBB) stained sodium dodecyl sulfate polyacrylamide gel electrophoresis (SDS-PAGE) gels of the purified Tde-SAS, Tde-SAS$_{1\text{-}246}$ and Tde-SAH proteins. **A**: Tde-SAS (elution volume 12.0 ml, calculated apparent molecular weight *ca*. 220 kDa, estimated multimericity: tetrameric). **B**: Tde-SAS$_{1\text{-}246}$ (elution volume 12.7 ml, calculated apparent molecular weight *ca*. 160 kDa, estimated multimericity: tetrameric). **C**: Tde-SAH (elution volume 15.5 ml, calculated apparent molecular weight *ca*. 44 kDa, estimated multimericity: dimeric). **D**. Domain mapping of Tde-SAS using limited proteolysis. CBB stained 12% SDS-PAGE gel showing quenched time-points taken from a reaction containing Tde-SAS and the non-specific protease subtilisin (0–150 mins). Two major fragments of *ca.* 26 kDa and *ca.* 21 kDa were generated, which respectively correspond to the N-terminal catalytic domain and C-terminal TPR-domain (as indicated by peptide mass fingerprinting, data not shown). See supplementary methods for further experimental details.

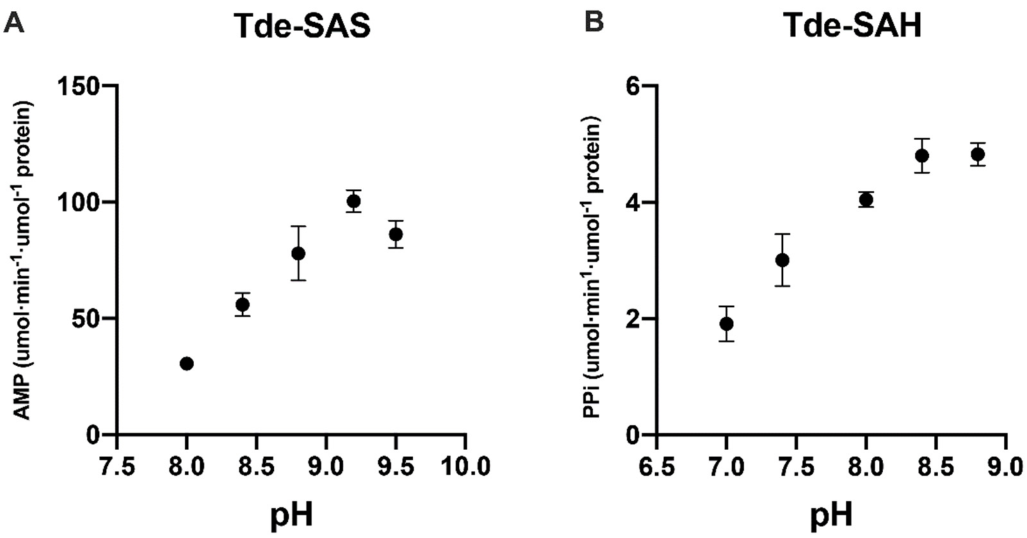

Figure S3. Optimal pH for ppGpp synthesis by Tde-SAS, optimal pH for ppGpp hydrolysis by Tde-SAH

A. Optimal pH for ppGpp synthesis by Tde-SAS. Y-axis: Specific rate of ppGpp synthesis plotted in units of micromoles of AMP (equimolar to ppGpp) formed per minute per micromole of Tde-SAS. X-axis: pH of reaction buffer (pH 8.0, 8.4, 8.8, 9.2, 9.5). **B**. Optimal pH for ppGpp hydrolysis by Tde-SAH. Y-axis: Specific rate of ppGpp hydrolysis plotted in units of micromoles of pyrophosphate formed per minute per micromole of Tde-SAH. X-axis: pH of reaction buffer (pH 7.0, 7.4, 8.2, 8.4, 8.8). Assays were performed in triplicate. Points plotted represent mean values with standard deviation shown in error bars. See supplementary methods for further experimental details

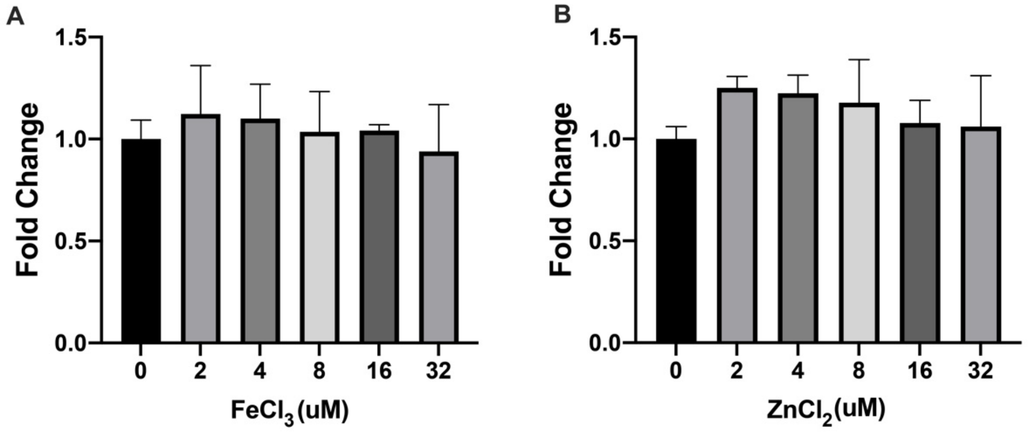

Figure S4. Effects of Fe^{3+} and Zn^{2+} ions on rate of ppGpp synthesis by Tde-SAS$_{1-246}$.

Plots respectively show the relative rate of ppGpp synthesis by Tde-SAS$_{1-246}$ in the presence of 0–32 μM Fe^{3+} ions (**Panel A**) or 0–32 μM Zn^{2+} ions (**Panel B**). The respective plots show the relative rate of ppGpp synthesis at a specific metal ion concentration normalized to the rate of ppGpp synthesis in the absence of added metal ion. All assays were performed in triplicate. Bars represent mean values with error bars indicating standard deviation. See supplementary methods for further experimental details.

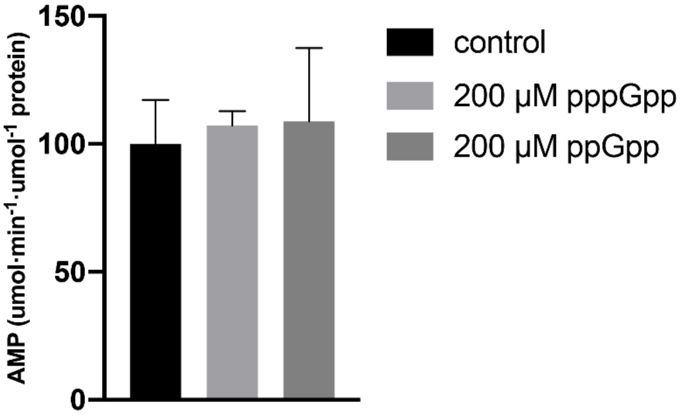

Figure S5. Rate of ppGpp synthesis by Tde-SAS with/without addition of 200 µM pppGpp or ppGpp.

Y-axis: Specific rate of ppGpp synthesis, plotted in units of micromoles of AMP (equimolar to ppGpp) formed per minute per micromole of Tde-SAS. Assays were performed in triplicate. X-axis. Bars represent mean values for rate of ppGpp synthesis by Tde-SAS in the absence of added (p)ppGpp (black, control), in the presence of 200 µM pppGpp (light gray), or in the presence of 200 µM ppGpp (dark gray). Error bars indicate standard deviation. See supplementary methods for further experimental details.

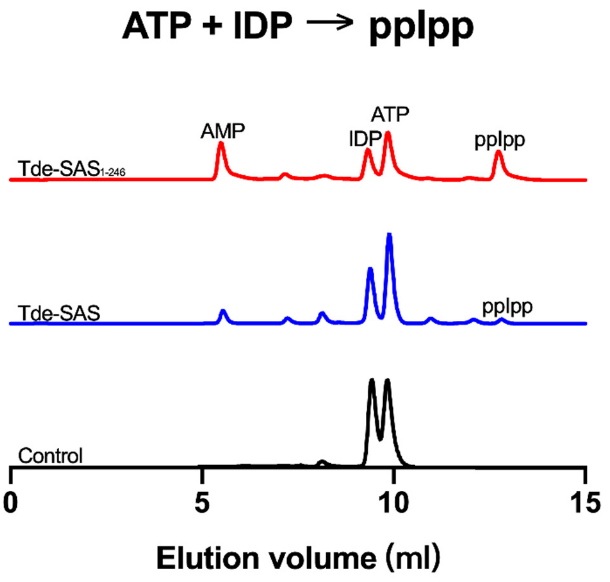

Figure S6. Tde-SAS$_{1\text{-}246}$ synthesizes the alarmone-like nucleotide ppIpp.
Representative chromatograms showing the respective product mixtures obtained after incubation of Tde-SAS$_{1\text{-}246}$ with adenosine 5'-triphosphate (ATP) and inosine 5'-diphosphate (IDP) (red chromatogram), Tde-SAS with ATP and IDP (blue chromatogram), and ATP + IDP with no enzyme (negative control, black chromatogram). Reaction products were identified by elution volume via comparison with previously-prepared standards. The peak corresponding to inosine 3',5'-bisdiphosphate (ppIpp) is indicated. Diphosphate is formed as a byproduct (data not shown).

	Genotype/Plasmid/Construct	Reference
Escherichia coli		
CF1648	Equivalent to MG1655 strain	(1)
CF1693	CF1648 Δ*relA251::*Km Δ*spoT207::*Cm	(1)
BL21 (DE3)	F-,*dcm, ompT, hsdS*(rB⁻, mB⁻), *gal,* λ(DE3)	Invitrogen
DH10B	F-,*mcr*A, Δ(*mrr*-*hsd*RMS-*mcr*BC), φ80*lac*ZΔM15,Δ*lac*X74, *rec*A1, *end*A1, *ara*D139, Δ (*ara*, *leu*)7697, *gal*U, *gal*K, λ-, *rps*L, *nup*G	Invitrogen
Treponema denticola	ATCC 35405^{T}	Lab stock
Fusobacterium nucleatum	ATCC 25586^{T}	Lab stock
**Plasmids**		
pET28a	Protein expression vector, P_{TAC} promoter, Kanamycin (Km) resistance	Novagen, Merck Millipore
pGEX	Protein expression vector, P_{TAC} promoter, Ampicillin (Amp) resistance	Novagen, Merck Millipore
pBAD33	Protein expression vector, P_{BAD} promoter, Chloramphenicol (Cm) resistance	(2)
pET28a-Tde-SAS	pET28a carrying full length Tde-SAS gene from *T. denticola* ATCC 35405 (cloned via BamHI/XhoI)	This work
pBAD33-Tde-SAS	pBAD33 carrying full length Tde-SAS gene from *T. denticola* ATCC 35405 (via SacI/SalI)	This work
pET28a-Tde-SAS_{1-246}	pET28a carrying the N-terminal catalytic domain of Tde-SAS (residues Met1- Gly246) (via BamHI/XhoI)	This work

pBAD33-Tde-SAS_{1-246}	pBAD33 carrying the N-terminal catalytic domain of Tde-SAS (residues Met1- Gly246) (via SacI/SalI)	This work
pET28a-Tde-SAH	pET28a carrying the SAH (TDE_RS08100) gene from *T. denticola* ATCC 35405 (cloned via BamHI/XhoI)	This work
pGEX-Tde-SAH	pGEX-4T1 carrying the SAH gene from *T. denticola* ATCC 35405 (via BamHI/XhoI)	This work
pET28a-Tde-SAH_{D74A}	pET28a carrying the SAH gene from *T. denticola* ATCC 35405 containing a D74A mutation (via BamHI/XhoI)	This work
pGEX-Tde-SAH_{D74A}	pGEX-4T1 carrying the SAH gene from *T. denticola* ATCC 35405 containing a D74A mutation (via BamHI/XhoI)	This work
pET28a-Fn-SAS	pET28a carrying the SAS (FN0926) gene from *F. nucleatum* ATCC 25586 (via BamHI/XhoI)	This work
pBAD33-Sa-RelP	pBAD33 carrying the RelP (NWMN_2405) gene from *Staphylococcos aureus* subsp. *aureus* str. Newman (via SacI/SalI)	(3)

Table S1. Bacterial strains and plasmids used in this study

Primer	Sequence (5'-3')	Description / Use
Tde-SAS-pET-Forward	ATCGCAGGATCC**ATG**TTGTCTGAAGACGTACC	Cloning Tde-SAS and Tde-SAS_{1-246} into pET28a
Tde-SAS-pET-Reverse	CGACCGTCCTCGAG**TTA**CACCATATCAATTTTTT	Cloning Tde-SAS into pET28a
Tde-SAS_{1-246}- pET-Reverse	ACGAGGTCGCTCGAG**TTA**TCCATGCTCAACATTATCAT	Cloning Tde-SAS_{1-246} gene into pET28a
Tde-SAS-pBAD-Forward	ATATGAGCTC**ATG**TTGTCTGAAGACGTACC	Cloning Tde-SAS and Tde-SAS_{1-246} into pBAD33
Tde-SAS-pBAD-Reverse	ATCGGTCGAC**TTA**CACCATATCAATTTTTTTTGCAA	Cloning Tde-SAS into pBAD33
Tde-SAS_{1-246}-pBAD-Reverse	ATATGTCGAC**TTA**TCCATGCTCAACATTATCAT	Cloning Tde-SAS_{1-246} into pBAD33
Sa-RelP-pBAD-Forward	ATATGAGCTC**ATG**TATGTAGATCGAAAACCATCAC	Cloning Sa-RelP into pBAD33
Sa-RelP-pBAD-Reverse	ATATGTCGAC**TTA**CTCTGTTATTTCAGAATGAA	Cloning Sa-RelP into pBAD33
Tde-SAH Forward	CTCGCACCATGGAAGGATCC**ATG**CTATCTTTTGATAGCAATAAGG	Cloning Tde-SAH into pET28a/pGEX-4T1
Tde-SAH Reverse	ATGGCACTCGAG**TTA**AAGCTTTCTTGCCTTGCGCATTT	Cloning Tde-SAH into pET28a/pGEX-4T1
Tde-SAH_{D74A} mutagenesis Forward	TCTTTAATCCAT**GCT**GTTATAGAAGATATC	Cloning Tde-SAH_{D74A} gene (GAT→GCT)
Tde-SAH_{D74A} mutagenesis Reverse	AGCCTTTAAAAGAACACTGTCGATGTA	Clone Tde-SAH_{D74A} gene
Fn-SAS Forward	CGCGGATCC**ATG**GATAAGCTAATAAAGGAAGAGTTTTT	Cloning Fn-SAS into pET28a
Fn-SAS Reverse	CCGCTCGAG**TTA**TTTAAATTTTATATCTAATTCTCTTTGGAC	Cloning Fn-SAS into pET28a

Table S2. Primers used in this study.

Supplementary Methods

Determination of multimericity

Size exclusion chromatography (SEC) was performed on a Superdex 200 10/300 GL column (GE Healthcare) connected to an AKTA purifier system (GE Healthcare), using gel filtration buffer (25 mM Tris-HCl pH 8.0, 500 mM NaCl, 1 mM EDTA, 5% glycerol), at a flow rate of 0.4 ml/min, monitoring the eluent at 280 nm. The protein molecular weight calibrants ferritin (440 kDa), aldolase (158 kDa), ovalbumin (44 kDa) and ribonuclease A (13.7 kDa) (GE healthcare) were used to construct a calibration curve (data not shown). Results are shown in **Figure S2.**

Domain mapping of the Tde-SAS protein using limited proteolysis

Tde-SAS (112 μg) was incubated with subtilisin (5.7 μg; Sigma) in 300 μl Tris-HCl pH 7.4 buffer at 37°C. Aliquots (18 μl) were removed at 0, 5, 10, 15, 30, 60 and 150 mins, immediately quenched by the addition of phenylmethylsulfonyl fluoride (PMSF; 2 mM; Sigma), before addition of (×5) SDS PAGE loading buffer, boiling for 10 mins, then analyzed on 12% SDS-PAGE gels, with Coomassie Brilliant Blue (CBB) staining. Bands were excised with a clean scalpel, and subjected to peptide mass fingerprint (PMF) analysis of trypsin-digested fragments at the Centre for PanorOmic Sciences (CPOS, The University of Hong Kong; Data not shown). A scanned image of the CBB stained SDS-PAGE gel is shown in **Figure S2.**

Determination of optimal pH values for ppGpp synthesis by Tde-SAS and ppGpp hydrolysis by Tde-SAH

Reactions were performed as described in the materials and methods section with minor modifications. **A**. Determination of the optimal pH for ppGpp synthesis by Tde-SAS. Reaction mixtures contained 50 mM Bis-Tris propane (pH 8.0, 8.4, 8.8, 9.2 or 9.5), 150 mM NaCl, 10 mM $MgCl_2$, 1 mM ATP, 1 mM GDP, 1 mM DTT and 250 nM Tde SAS protein. **B**. Determination of the optimal pH for ppGpp hydrolysis by Tde-SAH. Reaction mixtures contained 50 mM Tris-HCl pH (7.0, 7.4, 8.0, 8.4 or 8.8), 150 mM NaCl, 10 mM $MgCl_2$, 1 mM $MnCl_2$, 200 μM ppGpp and 250 nM Tde-SAH. Both sets of reaction mixtures were incubated at 37 °C. 20 μl aliquots of product mixtures were removed at 5, 10 and 15 min time points, and immediately quenched in liquid nitrogen for future analysis on a Mono Q 5/50 GL (1 mL) column. Results are shown in **Figure S3.**

Evaluation of rates of (pp)pGpp synthesis by Tde SAS/Tde $SAS_{1\text{-}246}$ in the absence/presence of 200 μM pppGpp or 200 μM ppGpp.

Reaction mixture A (100 μl) contained 50 mM Tris-HCl pH 8.8, 150 mM NaCl, 10 mM $MgCl_2$, 1 mM ATP, 1 mM GDP, 1 mM DTT. Reaction mixture B (100 μl) contained 250 mM Tris-HCl pH 8.8, 150 mM NaCl, 10 mM $MgCl_2$, 1 mM DTT, 50 nM Tde SAS or Tde $SAS_{1\text{-}246}$ protein, 200 μM ppGpp, 200 μM pppGpp, or no alarmone (control reaction). Reaction mixtures A and B were pre-incubated for 5 mins at 37 °C before mixing to initiate the reaction. At each time point (1, 2, 5, 10, 15, 20 and 30 mins), 20 μl aliquots of product mixture was removed, quenched by the addition of 2 mM EDTA (180 μl) and rapidly frozen in liquid nitrogen for subsequent analysis on a Mono Q column 5/50 GL (1 ml) as described in the materials and methods section. Results are shown in **Figure S5.**

Influence of Zn^{2+} or Fe^{3+} ions on ppGpp-synthesis activities of Tde-$SAS_{1\text{-}246}$.

Reactions were performed as described in the materials and methods section with minor modifications. Reaction mixtures containing 50 mM Tris-HCl pH 8.8, 150 mM NaCl,

10 mM $MgCl_2$, 1 mM DTT, 1 mM ATP, 1 mM GDP, $ZnCl_2$ (0, 2, 4, 8, 16 or 32 µM) or $FeCl_3$ (0, 2, 4, 8, 16 or 32 µM), and 10 nM Tde-SAS$_{1-246}$ were incubated at 37°C. Aliquots (20 µl) of product mixtures were removed at 5 min, 10 min and 15 min for analysis on a Mono Q column 5/50 GL (1 ml) as described in the materials and methods section. Results are shown in **Figure S4**.

Utilization of Mn^{2+}, Mg^{2+} Co^{2+} and other metal ions by Tde-SAH for ppGpp hydrolysis.

The rate of ppGpp hydrolysis by Tde-SAH in the presence of various metal ions was determined using a pyrophosphatase enzyme-coupled continuous fluorescent assay (EnzChek phosphate Assay Kit; Thermo Fisher Scientific, USA) as described in the materials and methods section with minor modifications. Reaction mixtures (200 µl) in 96-well plates contained 50 mM Tris-HCl pH 8.4, 150 mM NaCl, 1 mM DTT, 200 µM MESG, 0.2 Unit PNP, 200 µM ppGpp, 3 µM recombinant *Staphylococcus aureus* PpaC protein (Sa-PpaC, SAV1919; type II pyrophosphatase) (3), 200 µM ppGpp, 10 mM, 1 mM or 100 µM of the metal ion to be tested (aqueous solutions of the respective chloride salts added to the indicated concentration). Metal salts tested included: 10 mM $MgCl_2$, 1 mM $MnCl_2$, 10 mM $MgCl_2$ + 1 mM $MnCl_2$, 1 mM $CoCl_2$, 1 mM $CaCl_2$, 1 mM $NiCl_2$, 100 µM $FeCl_3$, 100 µM $ZnCl_2$. Assays were pre-incubated at 37 °C for 10 mins, then initiated by the addition of 250 nM Tde-SAH. Reactions were incubated at 37°C for 15 minutes with OD360 nm readings taken every 30s, using a SpectraMax M2e Multilabel Microplate Reader (Molecular Devices). Representative results are shown in **Figure 7.**

Determination of ppGpp and ppApp hydrolysis activities of Tde-SAH$_{D74A}$

Reactions were performed as described above with minor modifications. Reaction mixtures (200 µl) in 96-well plates contained 50 mM Tris-HCl pH 8.4, 150 mM NaCl, 1 mM DTT, 1 mM $MnCl_2$, 200 µM MESG, 0.2 Unit PNP, 3 µM Sa-PpaC, and 200 µM of ppGpp or200 µM of ppApp. Reactions were initiated by the addition of 250 nM Tde-SAH or Tde-SAH$_{D74A}$.

References

1. Xiao H, Kalman M, Ikehara K, Zemel S, Glaser G, Cashel M. Residual guanosine 3',5'-bispyrophosphate synthetic activity of *relA* null mutants can be eliminated by *spoT* null mutations. ***J Biol Chem*** 1991:266:5980-5990.
2. Guzman LM, Belin D, Carson MJ, Beckwith J. Tight regulation, modulation, and high-level expression by vectors containing the arabinose PBAD promoter. ***J Bacteriol*** 1995:177:4121-4130
3. Yang N, Xie S, Tang NY, Choi MY, Wang Y, Watt RM. The Ps and Qs of alarmone synthesis in *Staphylococcus aureus*. ***PLoS One*** 2019:14:e0213630.
4. Jimmy S, Saha CK, Kurata T, Stavropoulos C, Oliveira SRA, Koh A, Cepauskas A, Takada H, Rejman D, Tenson T, Strahl H, Garcia-Pino A, Hauryliuk V, Atkinson GC. A widespread toxin-antitoxin system exploiting growth control via alarmone signaling. ***Proc Natl Acad Sci USA*** 2020:117:10500-10510.
